# Mitochondrial ATP synthase c-subunit leak channel triggers cell death upon loss of its F_1_ subcomplex

**DOI:** 10.1038/s41418-022-00972-7

**Published:** 2022-03-23

**Authors:** Nelli Mnatsakanyan, Han-A Park, Jing Wu, Xiang He, Marc C. Llaguno, Maria Latta, Paige Miranda, Besnik Murtishi, Morven Graham, Joachim Weber, Richard J. Levy, Evgeny V. Pavlov, Elizabeth A. Jonas

**Affiliations:** 1 Department of Cellular and Molecular Physiology, The Pennsylvania State University College of Medicine, Hershey, PA USA; 2 Section of Endocrinology, Department of Internal Medicine, Yale University, PO Box 208020, New Haven, CT USA; 3 Department of Human Nutrition & Hospitality Management, University of Alabama, Tuscaloosa, AL USA; 4 Department of Anesthesiology, Guizhou Provincial People’s Hospital, Guiyang, Guizhou China; 5 Center for Cellular and Molecular Imaging, Yale University, New Haven, CT USA; 6 Center for Neural Science, New York University, New York, NY USA; 7 Texas Tech University, Department of Chemistry and Biochemistry, Lubbock, TX USA; 8 Department of Anesthesiology, Columbia University Medical Center, New York, NY USA; 9 Department of Basic Sciences, New York University, New York, NY USA

**Keywords:** Cell biology, Neuroscience

## Abstract

Mitochondrial ATP synthase is vital not only for cellular energy production but also for energy dissipation and cell death. ATP synthase c-ring was suggested to house the leak channel of mitochondrial permeability transition (mPT), which activates during excitotoxic ischemic insult. In this present study, we purified human c-ring from both eukaryotic and prokaryotic hosts to biophysically characterize its channel activity. We show that purified c-ring forms a large multi-conductance, voltage-gated ion channel that is inhibited by the addition of ATP synthase F_1_ subcomplex. In contrast, dissociation of F_1_ from F_O_ occurs during excitotoxic neuronal death suggesting that the F_1_ constitutes the gate of the channel. mPT is known to dissipate the osmotic gradient across the inner membrane during cell death. We show that ATP synthase c-subunit knock down (KD) prevents the osmotic change in response to high calcium and eliminates large conductance, Ca^2+^ and CsA sensitive channel activity of mPT. These findings elucidate the gating mechanism of the ATP synthase c-subunit leak channel (ACLC) and suggest how ACLC opening is regulated by cell stress in a CypD-dependent manner.

## Introduction

Mitochondrial F_1_F_O_ ATP synthase is responsible for ATP synthesis during oxidative phosphorylation and is one of the most abundant proteins in the mitochondrial inner membrane. ATP synthase is a multi-subunit complex consisting of the membrane-embedded F_O_ and hydrophilic F_1_ subcomplexes. The peripheral and central stalks of ATP synthase link F_O_ with F_1_ and enhance the catalytic activity and chemo-mechanical coupling of ATP synthase [1].

ATP synthase was shown to contribute to energy dissipation and initiation of cell death through the formation of an uncoupling channel within its c-ring [2–4] or between ATP synthase dimers [5–10]. A model for permeability transition pore opening was suggested recently based on the structural studies of ovine ATP synthase [11]. Cryo-EM maps of ATP synthase exposed to calcium revealed conformational changes within the ATP synthase, including a retracted conformation of subunit e and disassembled c-ring, failing to demonstrate a pore, partially due to the structural determination of protein in detergent [11]. While structural studies can provide valuable information about different conformational snapshots of ACLC on the atomic level, electrophysiological studies are crucial for characterizing the biophysical properties of the channel, defining the gating mechanism of ACLC and its role in mPTP formation. Here we have applied a multidisciplinary approach to characterize the ACLC. We came up with the following set of criteria, in evaluating whether any ATP synthase subunit can form a channel of the mPT: 1) The pore-forming subunit(s) of any ion channel, including the mitochondrial permeability transition pore (mPTP), should form a channel without associated regulatory subunits in its purified form. 2) mPTP is a voltage-gated channel, which means that its channel activity should be modulated in response to changes in transmembrane voltage. 3) Activation and inactivation of ion channels are determined by gating of the channel which regulates the passage of ions. 4) Genetic ablation of the mPTP-forming protein will eliminate its known high (~1.5 nS) conductance activity [12].

In this current report we have addressed these criteria with the following findings: 1) We show that human ATP synthase c-ring forms a large conductance (~1.5 nS) and 2) voltage-gated channel in its purified form without any regulatory subunits. 3) We show that the ATP synthase F_1_ subcomplex forms a gate of c-ring channel by inhibiting its activity. In support, we show that F_1_ disassembles from F_O_ in neurons exposed to glutamate toxicity and that this dissociation is sensitive to CsA, a well-studied inhibitor of CypD-dependent mPT. These data suggest that conformational changes of F_1_ towards F_O,_ or the complete dissociation of these two subcomplexes occurs during permeability transition and ACLC opening. In keeping with a central role for ACLC in mPT 4) we demonstrate that significant knockdown (85%) of c-subunit genes eliminates high conductance mPTP-like openings. These findings suggest that the F_1_ constitutes a gate of ACLC and that the c-ring leak channel comprises a main pore-forming unit of the highly regulated CypD-dependent mPT.

## Results

### ATP synthase c-ring purified from different hosts forms a large multi-conductance voltage-gated channel sensitive to ATP

**Fig. 1:**
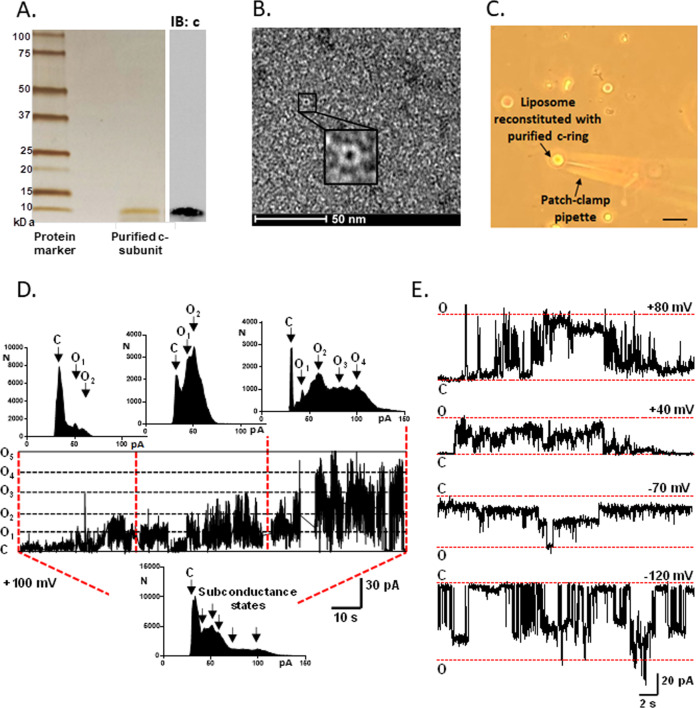
Human ATP synthase c-subunit purified from different hosts forms a large multi-conductance voltage-gated channel. **A** Left panel: Silver-stained gel of purified c-subunit from HEK 293 cells. The bands on the first lane of the gel represent Precision Plus Protein Standards marker, the second lane was left empty, the single band at ~10 kDa position on the third lane represents purified c-subunit. The sample was resolved by SDS-PAGE. Right panel: Immunoblot analysis of purified protein (representative of *n* = 3 blots); the membrane was probed with anti-c-subunit antibody. **B** EM image of the purified and negatively stained c-subunit ring in 0.05% n-dodecyl β-D-maltoside (DDM). 2% uranyl acetate was used to stain the purified protein. Inset shows higher magnification of purified c-ring. **C** Photomicrographic image during a patch-clamp recording showing the patch-clamp pipette sealed onto a liposome reconstituted with the purified c-ring. Scale bar, 50 µm. **D** Representative excised patch proteoliposome recording of c-ring (HEK 293) at +100 mV holding voltage. Eleven consecutive traces were concatenated and used for amplitude histogram analysis. The amplitude histogram of c-ring channel activity demonstrates subconductance states of ∼70–100 pS, 300 pS, 600 pS, 700 pS and a presumed fully open state of 1100 pS. Conductances are based on the assumption of a linear current-voltage relationship at +100 mV. C closed, O1–5, open. **E** Representative lipid bilayer recordings of the human c-ring channel purified from *E. coli* at different voltages from different experiments.

To study the biophysical characteristics of the c-ring as a pore-forming component of mPTP, we purified human c-ring from HEK 293 cells (Fig. 1A). We have optimized the c-subunit overexpression and purification procedure reported previously [3] to increase the purity of the preparation. Shown in Fig. 1A is a silver-stained gel of this purified protein which illustrates that it is free from contamination by other proteins. The purified c-subunit is also not contaminated with ATP synthase F_1_ (Supplementary Fig. S1A). We confirmed the preservation of the native ring structure of purified c-subunit with negative stain EM studies (Fig. 1B) and clear native PAGE (Supplementary Fig. S1B).

To further rule out the possibility of purified c-ring contamination with other mitochondrial proteins, we heterologously overexpressed and purified the human c-ring from *E. coli*. Human ATP synthase c-subunit is encoded by three different genes ATP5G1, ATP5G2, and ATP5G3. Each of the three genes has a distinct mitochondrial targeting sequence but encodes the identical 75-amino acid-long mature protein. Here in this study, the sequence of human ATP synthase c-subunit corresponding to the mature protein was cloned into the bacterial pEX-1 expression vector. DNA codon optimization strategy was used to increase the expression level of protein in *E. coli* cells (Supplementary Fig. S1C). The protein was tagged with six histidine residues at its C-terminus to facilitate purification (Ni-NTA). Interestingly, overexpressed mature c-subunit traffics into *E. coli* membranes (Supplementary Fig. S1D), therefore the N-terminal sequence of c-subunit seems to be dispensable for membrane targeting in *E. coli*. We found that our preparation of c-subunit purified from *E. coli* is not contaminated with ATP synthase F_1_ (Supplementary Fig. S1E). The purified c-subunit demonstrated the same oligomeric composition as the c-subunit purified from HEK 293 cells as assessed by clear native page (Supplementary Fig. S1B). In both cases in the absence of denaturation with SDS, human c-subunit was detected at ∼250 kDa, suggesting the presence of tetramers of octameric rings formed by purified c-subunits, as has been observed previously [3, 13]. This is also in agreement with the recent report of the cryo-EM structure of porcine ATP synthase tetramers [14]. Partial to complete disassembly of higher-order oligomeric states of c-ring was observed in the presence of SDS as demonstrated by the presence of bands at ~66 and ~8 kDa positions (Supplementary Fig. S1B). There is remarkable variability in c-ring oligomeric composition in different species with c-rings composed of between 8–17 subunits [15–17] and the determining factor for the ring stoichiometries is likely to be the amino acid sequence of the protein [18], as we observed here in the case of human c-subunit overexpression in *E. coli*.

Next, we set out to investigate the single-channel activity of purified c-rings reconstituted in liposomes (Fig. 1C, D). Shown in Fig. 1D is a patch-clamp recording of purified c-ring (HEK 293) reconstituted in liposomes. The current recordings demonstrate large multi-conductance channel activity with an average peak conductance of 1.1 nS (Fig. 1D) similar to previously published data [3, 19]. We concatenated eleven consecutive traces (10-second long) of channel activity for amplitude histogram analysis. The amplitude histogram demonstrates subconductance states of 70–100 pS, 300 pS, 600 pS, 700 pS and a presumed fully open state of 1100 pS (Fig. 1D). Data shown here are consistent with a continuously expanding pore such as reported previously for the mitochondrial megachannel [19]. Similar low and high conductance states were reported for mPTP during electrophysiological recordings of isolated mitochondria [12, 20].

We also characterized the single-channel activity of the human c-ring purified from *E. coli* by performing planar lipid bilayer recordings (Fig. 1E, Supplementary Fig. S1G–I). Shown in Fig. 1E are lipid bilayer recordings at different voltages from different experiments. The group data of single-channel recordings demonstrate that c-ring purified from either HEK 293 or *E. coli* forms voltage-gated channel activity with a similar peak conductance and probability of opening (NPo; see methods) (Supplementary Fig. S1H, I). Control recordings were performed in the presence of membrane scaffold protein, MSP (Supplementary Fig. S1J) to show that a known non-channel forming protein fails to produce ion channel activity. Shown in Supplementary Fig. S1F is a silver-stained gel of the purified MSP that was used for recordings. Control recordings were also performed with empty bilayers (Supplementary Fig. S1K) to rule out the possibility of membrane leak formation due to the presence of residual detergent or physical rupture of the membrane during recordings.

**Fig. 2:**
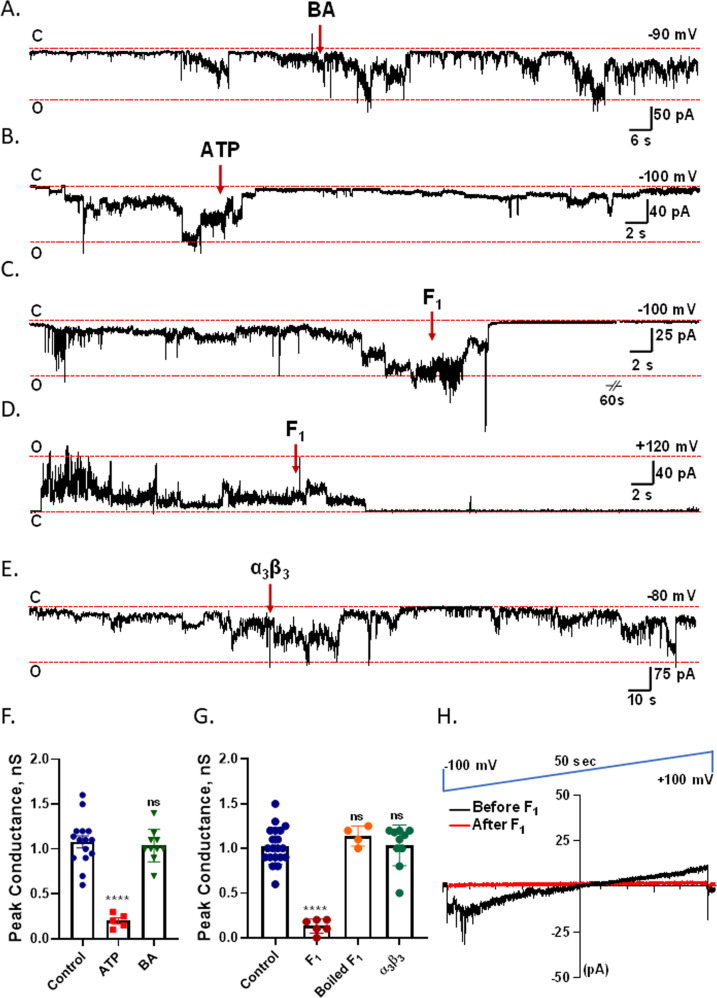
ACLC is inhibited by ATP and gated by F_1_. **A**, **B** Representative lipid bilayer recordings of human c-ring before and after the addition of ANT inhibitor bongkrekic acid (BA, 5 µM, −90 mV holding voltage) and ATP (1 mM, −100 mV holding voltage). C refers to closed state of the channel activity, O refers to open state of channel activity. **C** Representative trace of an excised proteoliposome patch recording of c-ring before and after the addition of F_1_ (5 µg, final concentration, −100 mV holding voltage). **D** Representative lipid bilayer recordings of c-ring before and after the addition of F_1_ (5 µg, +120 mV holding voltage) or **E** α_3_β_3_ complex (5 µg, −80 mV holding voltage). **F** Group data for peak conductances of c-subunit lipid bilayer recordings in response to ATP (*n* = 5) or BA (*n* = 10). ****P < 0.0001 (paired *t*-test), in which each channel recording was compared before and after reagent addition. For simplicity of presentation, the control data are grouped in the same bar. **G** Group data for peak conductances of c-subunit lipid bilayer recordings in response to purified F_1_ (*n* = 6), boiled F_1_ (*n* = 4) and α_3_β_3_ complex (10); ****P < 0.0001, paired *t*-test; as in **F** the control data from each paired study are grouped into one bar for simplicity of presentation. **H** Representative excised patch proteoliposome recordings of the c-subunit channel during continuous ramp voltage clamp from −100 mV to +100 mV before and after F_1_ addition.

To determine possible contamination of purified c-subunit with other channel-forming mitochondrial proteins, such as the adenine nucleotide carrier (ANT), we have carried out channel recordings of human c-ring (purified from HEK 293 cells) in the presence of bongkrekic acid (BA), a specific inhibitor of ANT [21]. Figure 2A, F show that c-ring channel activity is not affected by the addition of BA during the channel recordings, confirming a lack of contamination with ANT.

One of the biophysical characteristics of the mPTP is its sensitivity to adenine nucleotides [20, 22]. We have reported previously that purified ATP synthase from porcine heart mitochondria and c-subunit (purified from HEK 293 cells) both demonstrate ATP-sensitive channel activities with different binding affinities [3, 4]. Here, we show that ATP addition inhibits channel activity during a continuous bilayer recording of the human c-ring (purified from *E. coli*) (Fig. 2B, F). In a previous report, we showed that channel activity was equally attenuated by ATP, ADP and AMP, suggesting that binding of these adenine nucleotides rather than ATP hydrolysis is required for channel inhibition [3]. We have previously shown that another well-known inhibitor of mPTP, CsA, fails to inhibit the c-ring channel activity [3] that lacks F_1_ and stator components, since the binding site for its interacting partner, CypD, is located in the OSCP subunit [10, 23].

### ACLC is gated by purified F_1_

Mitochondrial ATP synthase consists of two structural domains, hydrophilic F_1_ located in the matrix and membrane-embedded F_O_, which are linked together by the central and peripheral stalks. According to high-resolution structures of ATP synthase, the central stalk subunits of F_1_, γ, δ, and ε interact with the c-ring [14]. We have shown earlier that separation of F_1_ from F_O_ occurs in isolated mitochondria during exposure to high Ca^2+^ that induces the mPTP opening [3] suggesting that F_1_ could constitute a channel gate and that conformational changes in ATP synthase are needed to open the channel. Here we set out to study if the application of purified F_1_ during channel recordings will inhibit the voltage-dependent c-ring channel activity. Supplementary Fig. S2A, B demonstrate the SDS-PAGE and immunoblot analysis of purified F_1_ respectively. Figure 2C demonstrates a continuous patch-clamp recording of c-ring reconstituted proteoliposomes. The addition of purified F_1_ to the bath caused the inactivation of the channel (Fig. 2C, G). Ramp voltage recordings before and after the addition of F_1_ show that channel conductance is inhibited by F_1_ at all voltages between −100 mV and +100 mV (Fig. 2H). To confirm these findings, we studied if F_1_ application would inhibit the c-ring channel activity during planar lipid bilayer experiments. Indeed, we observed channel inactivation upon F_1_ application (Fig. 2D, Supplementary Fig. S2C). In contrast to the inhibition found after F_1_ addition, the addition of ATP synthase α_3_β_3_ complex, lacking the central stalk subunits gamma, delta and epsilon (Supplementary Fig. S2D) did not inhibit the channel activity (Fig. 2E, G) suggesting that specific interactions between the central stalk subunits gamma, delta and epsilon with the c-ring are necessary for channel inhibition with F_1_. We also show that the addition of boiled (denatured) F_1_ during the recordings did not inhibit the c-ring channel activity (Fig. 2G), These findings suggest that F_1_ forms a gate of the ACLC.

### Dissociation of ATP synthase F_1_ from F_O_ occurs during excitotoxic neuronal death

Our findings thus far show that ATP synthase c-ring demonstrates voltage dependent single-channel activity in the absence of F_1_ and that the addition of F_1_ inhibits the channel activity. We, therefore investigated how pathological events might alter the oligomeric state of ATP synthase, its assembly, and the F_1_/F_O_ stoichiometric ratio. The membrane-embedded c-ring is part of the ATP synthase F_O_ subcomplex. Tight interaction between F_1_ and F_O_ subcomplexes, and efficient chemo-mechanical coupling, have been suggested to be crucial for efficient ATP synthesis [24].

**Fig. 3:**
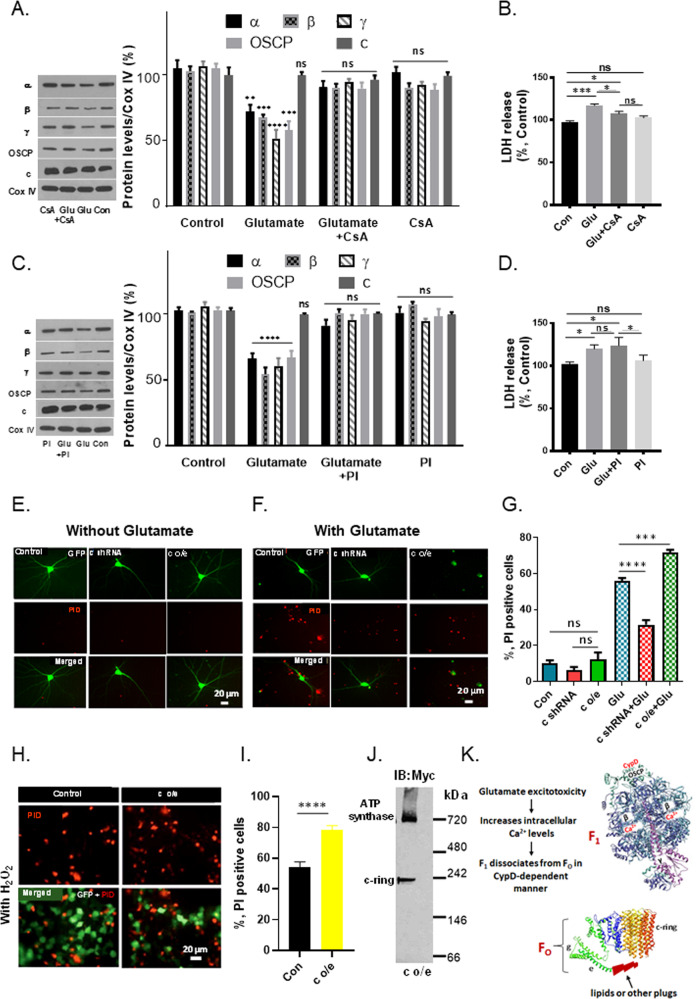
Dissociation of ATP synthase F_1_ from F_O_ occurs under glutamate excitotoxic conditions. **A**, **C** Immunoblot analysis of mitochondria isolated from hippocampal neurons before and after glutamate treatment in the absence or presence of CsA (*n* = 3 independent cultures, **P < 0.0017, ***P < 0.0003, ****P < 0.0001) or the absence or presence of a proteasome inhibitor (PI) (*n* = 3 independent cultures, ****P < 0.0001), respectively, one-way ANOVA Dunnett’s multiple comparisons test. These data confirm that F_1_ separates from c-subunit, then F_1_ components are proteolyzed. **B**, **D** Group data for cell death (lactate dehydrogenase (LDH) release) of hippocampal neurons under the indicated conditions. CsA, but not proteasome inhibitor (PI), inhibits cell death by preventing F_1_/F_O_ dissociation. For CsA data: *n* = 3 independent cultures, ***P = 0.0007 for Con vs. Glu; *P = 0.0377 for Con vs. Glu+CsA; *P = 0.0434 for Glu vs. Glu+CsA, **P = 0.0053 for Glu vs. CsA. For proteasome inhibitor (PI) group data: *n* = 3 independent cultures, *P = 0.0376 for Con vs. Glu; *P = 0.0135 for Con vs. Glu+PI; *P = 0.0376 for Glu+PI vs. PI. Statistics for group data were calculated using one-way ANOVA Dunnett’s multiple comparisons test. Error bars refer to SEM. Antibodies for different ATP synthase subunits were used as indicated. **E**, **F** Images of propidium iodide (PID) staining of cultured hippocampal neurons before and after glutamate treatment (cells were treated with 20 μM glutamate at DIV 12–14 after transfection and stained with propidium iodide 18 h after glutamate stimulation). Primary hippocampal neurons express GFP, GFP plus c-subunit shRNA or GFP plus human c-subunit. Green: GFP; Red: PID. **G** Group data of propidium iodide staining of cultured hippocampal neurons, expressing the indicated constructs with or without glutamate treatment. 65–106 micrographs were used, *n* = 3–5 independent cultures, ***P = 0.0004, ****P < 0.0001, one-way ANOVA. Glutamate produces substantial cell death, which is increased by c-subunit overexpression and inhibited by knockdown of c-subunit with shRNA. The histogram represents the death of only GFP-expressing cells. **H**, **I** Images and group data of cell death measured with propidium iodide for HEK 293 cells transfected with constructs expressing GFP or GFP plus human c-subunit constructs, measured at 24 h after a 30 min. exposure to 1 mM H_2_O_2_ (10–23 wells under each condition, ***p < 0.0001, unpaired t-test). Green: GFP; Red: PID. c-subunit overexpression substantially increases cell death compared with H_2_O_2_ treatment alone. The histogram represents cell death of only GFP-expressing cells. **J** Immunoblot analysis after non-denaturing Blue Native Page (BNP) of mitochondria isolated from HEK 293 cells overexpressing Myc-tagged human c-subunit. Antibody for Myc-tag was used. c-subunit is only partially assembled in ATP synthase, detected as a band ~720 kDa position, while the band at ~242 kDa represents the higher-order c-ring oligomer uncomplexed with F_1_. The gel is representative of 3 gels. **K** Schematic representation of non-reversible dissociation of ATP synthase F_1_ from F_O_ during glutamate-induced excitotoxic conditions. ATP synthase subunits are drawn as ribbon representations (modified PDB ID code: 6J5I) [14].

To determine if excitotoxicity produces dissociation of F_1_ from F_O_ and induces any changes in ATP synthase oligomeric state in a pathophysiological setting, we exposed primary hippocampal and cortical neurons to glutamate toxicity. After 18 h of glutamate exposure, mitochondria were isolated and F_1_ and F_O_ components were analyzed by immunoblotting. The levels of F_1_ subunits alpha, beta, gamma and OSCP were significantly reduced compared to those of vehicle-exposed neurons in both hippocampal (Fig. 3A) and cortical neurons (Supplementary Fig. S3A). In contrast, the level of c-subunit was not decreased, suggesting a marked change in F_1_/c stoichiometry that enhanced the level of “free” c-rings not complexed with F_1_. High levels of free c-ring in mitochondrial membranes could increase the probability of c-ring channel opening and predispose neurons to death in a voltage dependent manner. To determine if dissociation of F_1_ from F_O_ is associated with the onset of mPTP-induced cell death, we used CsA, the well-known inhibitor of CypD that prevents mPTP opening by binding within the peripheral stalk [23]. We found that CsA inhibits the reduction of F_1_ subunit levels and therefore prevents changes in F_1_/c stoichiometry under glutamate excitotoxic conditions (Fig. 3A), presumably by preventing conformational changes within the ATP synthase that result in the separation of F_1_ from c-ring. CsA also prevents cell death under these conditions as observed in studies of lactate dehydrogenase (LDH) release (Fig. 3B). To further study the nature of F_1_/F_O_ dissociation and the disappearance of F_1_ components, we treated neurons with glutamate in the presence of the proteasome inhibitor (PI) MG-132. This treatment prevented the loss of F_1_ components, by preventing the degradation of F_1_ after glutamate exposure (Fig. 3C). However, it failed to prevent cell death measured by LDH release into the medium (Fig. 3D), suggesting that CsA protects against dissociation of F_1_ from F_O_ whereas the proteasome inhibitor only prevents the degradation of F_1_ subunits without preventing their dissociation from F_O_.

To study the changes in the oligomeric state of mitochondrial ATP synthase under conditions of glutamate toxicity, we analyzed glutamate-treated neuronal mitochondria with Blue Native Page (BNP). We found that glutamate induced the breakdown of ATP synthase dimers into monomers (Supplementary Fig. S3B, left panel), which is in agreement with a recent report [25]. We also observed an overall increase of free c-subunit levels in mitochondrial membranes in digitonin or n-dodecyl β-D-maltoside (DDM) solubilized mitochondria under conditions of glutamate toxicity, whereas the level of free, uncomplexed F_1_ was reduced (Supplementary Fig. S3B), consistent with the SDS PAGE studies.

These data suggest that reduced F_1_/c stoichiometric ratio and increased amount of free c-ring dissociated from F_1_ are observed under glutamate excitotoxic conditions in which CsA-sensitive mPTP opening occurs.

To investigate further the role of free c-ring in the onset of mPT, we measured provoked cell death in primary hippocampal neurons (Fig. 3E–G) and HEK 293 cells (Fig. 3H, I) after overexpressing c-subunit. We co-expressed GFP with the c-subunit since they have a high co-expression level, therefore indicating that GFP transfected cells represent c-subunit transfected cells. Cell death was measured only in GFP-expressing cells (Fig. 3E–I). After confirming that overexpressed c-subunit co-localizes with mitochondria in primary hippocampal cultures (Supplementary Fig. S3C), we determined that overexpression of c-subunit alone under these conditions does not induce death, consistent with our previous studies [26] but that it aggravates glutamate-induced cell death (Fig. 3E–G), supporting its role in mPT. In contrast, reduction of c-subunit levels by shRNA prevents cell death under glutamate excitotoxic conditions (Fig. 3E–G), suggesting that F_1_/F_O_ dissociation and the change in F_1_/F_O_ stoichiometry precedes the cell death.

Similarly, overexpression of c-subunit in HEK 293 cells markedly increased H_2_O_2_-induced cell death (Fig. 3H, I). Immunoblot analysis of mitochondria isolated from c-subunit-overexpressing HEK 293 cells revealed that c-subunit is only partially assembled with the ATP synthase complex (Fig. 3J). A substantial amount of free c-ring uncomplexed with F_1_ is present in mitochondrial membranes (Fig. 3J; the band at ~242 kDa position is free c-subunit complex, the band at ~720 kDa position is ATP synthase). Taken together, the data show that the stochiometric change in F_1_/c ratio precedes cell death in response to stimuli that induce mPT, but that c-subunit expression in the absence of a death stimulus does not depolarize the inner membrane.

Death of glutamate-treated neurons was additionally assessed by staining with Annexin V and propidium iodide (PID) to differentiate apoptotic cell death from the total, as previously described [27]. Annexin V and PID staining of hippocampal neurons are shown in (Supplementary Fig. S3D–F). These data suggest that apoptotic death comprises at least 50% of the total death. The initial events in mPT before cell death include mitochondrial inner membrane depolarization. A decrease of the inner membrane potential was observed under glutamate excitotoxic conditions (Supplementary Fig. S3G, H), and this was inhibited by CsA, confirming the role of loss of mitochondrial membrane potential in glutamate excitotoxicity in mPTP-induced neuronal death as described previously [28, 29].

We have previously studied the detachment of F_1_ subcomplex from c-ring in response to Ca^2+^ treatment in isolated mitochondria by immunoprecipitation [3]. Our studies revealed that upon Ca^2+^ induced mitochondrial swelling the release of c-ring oligomers occurred, which was inhibited by CsA and ADP treatment. Furthermore, as expected, detachment of F_1_ from c-subunit was not observed in mitochondria from CypD KO animals [3], suggesting that CypD-mediated Ca^2+^ binding to F_1_ destabilizes ATP synthase causing detachment of F_1_ from c-subunit, which initiates mPTP.

Our findings suggest that F_1_ constitutes an important inactivation gate of the ACLC (Fig. 3K) and small changes in the F_1_/c stoichiometry alter inner membrane permeability after a cell death stimulus by increasing the open probability of ACLC and enhancing the risk of neuronal death.

### ATP synthase is required for calcium-induced osmotic changes underlying permeability transition

**Fig. 4:**
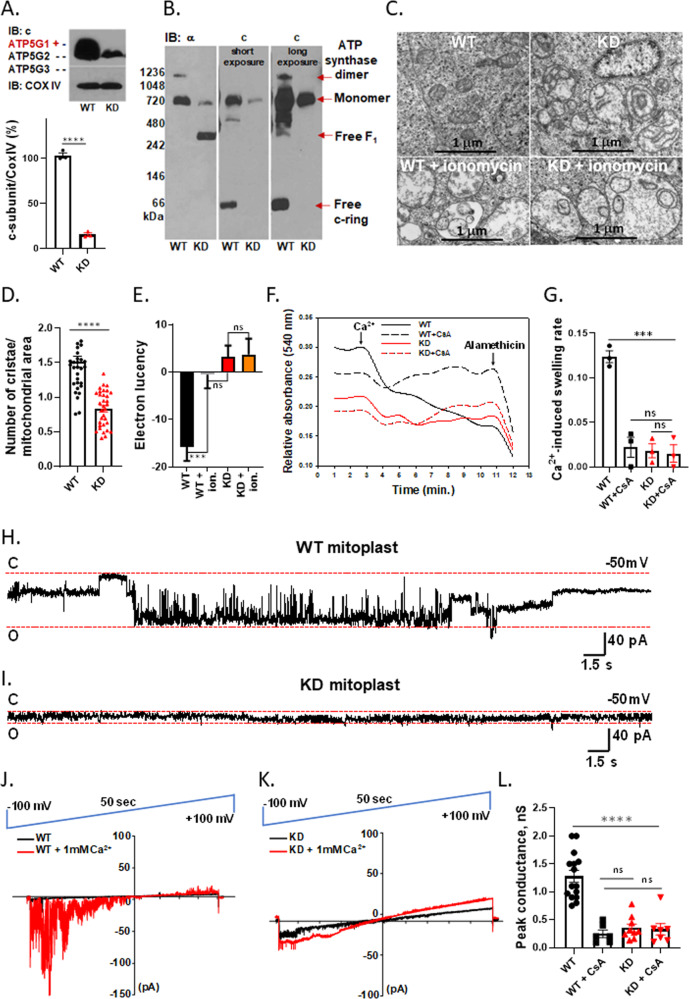
ATP synthase is required for normal mitochondrial cristae morphology and large-conductance mPTP-like channel activity. **A** Representative western blot of WT and c-subunit KD mitochondria. Group data show that only 15% c-subunit is expressed in KD. (*n* = 4), ****P < 0.0001, unpaired *t*-test. **B** Immunoblot after non-denaturing Blue Native Page (representative of *n* = 3). Free c-subunit is readily discernible in WT but not in KD mitochondria. **C** EM images of WT and KD mitochondria before and after treatment with ionomycin (mouse ES cells (ESCs) were treated with 1 µM ionomycin for 20 min before they were fixed for further EM analysis). Swelling (electron lucency) observed in WT ESC mitochondria is not present in c-subunit KD ESCs mitochondria. **D** Group data of the total number of mitochondrial cristae divided by the area of mitochondria (*n* = 34 mitochondria for WT, *n* = 39 for KD, (****P < 0.0001, unpaired *t*-test). **E** Group data showing electron lucencies before and after treatment with ionomycin, (***P < 0.0004, *n* = 11 micrographs for WT, *n* = 11 for WT + ionomycin, *n* = 15, for KD, *n* = 15, for KD + ionomycin). **F** c-subunit KD ESC isolated mitochondria do not swell after Ca^2+^ addition. Representative traces for WT and KD isolated mitochondrial swelling, monitored upon Ca^2+^ (0.5 mM) addition in the presence or absence of CsA. CsA-sensitive swelling observed in WT mitochondria is not present in c-subunit KD mitochondria. The application of the membrane permeabilizing agent, alamethicin (10 μM), revealed that KD mitochondria were otherwise intact and capable of swelling. **G** Histogram for group data representing the Ca^2+^-induced swelling rate. Data show mean ± SEM, *n* = 3; ***P = 0.0002, One-Way ANOVA with multiple comparison test. **H**, **I** Representative patch-clamp recordings of WT and KD mitoplasts, pre-treated with Ca^2+^ (1 mM) at −50 mV holding voltage. **J**, **K** Representative recordings of WT and KD mitochondria during continuous ramp voltage clamp from −100 mV to +100 mV in the presence or absence of Ca^2+^ (1 mM). **L** Group data for peak conductances of mitoplasts, pre-treated with 1 mM Ca^2+^ in the presence or absence of CsA (5 uM). The Ca^2+^ and CsA-sensitive activity of WT mitoplasts is absent in c-subunit KD mitoplast recordings. A paired *t*-test was used to compare WT or KD recordings with and without CsA (5 uM). Unpaired *t*-test was used to compare WT and KD recordings (*n* = 15 for WT, *n* = 9 for KD, *n* = 6 paired patches for WT + CsA, *n* = 7 paired patches for KD + CsA ****P < 0.0001).

Our studies so far suggest that free c-ring dissociated from F_1_ is responsible for the large conductance channel activity associated with pathological PT. Pathological PT is associated with water accumulation in the mitochondrial matrix after a high calcium load. Therefore, to determine if free c-subunit is required for calcium-dependent matrix swelling, we used CRISPR-Cas9 to knockdown c-subunit expression in mouse embryonic stem cells (ESCs). We successfully deleted five out of six alleles of the three genes (ATP5G1, ATP5G2 and ATP5G3) encoding ATP synthase c-subunit. The remaining one ATP5G1 allele resulted in 15 percent expression of c-subunit in mitochondrial inner membranes (Fig. 4A). Blue Native PAGE confirmed the existence of ATP synthase dimers, monomers, as well as free c-ring in WT ESCs (Fig. 4B). In contrast, c-subunit KD cells failed to form ATP synthase dimers, had low monomer content and no free c-ring (Fig. 4B). Interestingly, the free c-ring is present in native mitochondrial membranes waiting to be assembled with F_1,_ but it is completely absent in KD, which had severely reduced ATP synthase levels. In contrast, free F_1_ is present in KD mitochondria but it is absent in WT (Fig. 4B). ESCs in general have low coupled oxygen consumption [30]. Nevertheless, we show that oxygen consumption and membrane potential of c-subunit KD mitochondria were reduced compared to WT, confirming the contribution of the c-subunit to the ESCs mitochondrial leak and the inability of c-subunit KD cells to perform normal oxidative phosphorylation due to the absence of fully assembled ATP synthase (Supplementary Fig. S4A).

The formation of ATP synthase dimers is crucial for normal mitochondrial morphology and for the biogenesis of mitochondrial cristae, which are heavily folded mitochondrial inner membrane structures designed to increase the surface for oxidative phosphorylation [31, 32]. To assess the mitochondrial morphology in KD ESCs that completely lack ATP synthase dimers, we performed electron microscopy (EM) analysis. We found that KD mitochondria had unusual cristae morphology with onion-like circular structures (Fig. 4C). There was also a significant decrease in the number of cristae per unit area of mitochondria (Fig. 4D). A traditional assay for mPT is swelling as assessed by EM criteria in cells [33]. Mitochondrial swelling in the WT ESCs was observed upon the addition of ionomycin, which increases cellular Ca^2+^ concentration (Fig. 4C, E). Using EM criteria, KD mitochondria appeared swollen before the addition of ionomycin to the cells. The addition of ionomycin to KD ESCs produced no additional swelling by EM measurements (Fig. 4C, E), suggesting the absence of Ca^2+^-induced mPT. To show this in a different assay, we used light scattering measurements of isolated mitochondria (Fig. 4F, G), which confirmed the findings in the ESCs, suggesting that isolated KD mitochondria are partially swollen at rest and that the addition of Ca^2+^ causes little additional swelling as compared to the robust response observed in isolated WT mitochondria (Fig. 4C, E–G). CsA prevented swelling in WT but had no effect on KD mitochondria, suggesting a lack of CypD-dependent mPT in KD mitochondria. The application of the membrane permeabilizing agent, alamethicin, confirmed that KD mitochondria, like WT, were still capable of further swelling and that the low rates of Ca^2+^-induced swelling were in fact due to reduced level of c-subunit (Fig. 4F, G).

These findings suggest that the ATP synthase is required for normal and pathological mitochondrial osmotic regulation.

### c-subunit gene KD decreases mPTP-like channel activity of mitochondria

The mPTP was first characterized as an abnormal swelling of mitochondria upon high calcium overload [34]. Later, it was shown by electrophysiological recordings that the calcium-induced mitochondrial swelling was due to the opening of a high conductance, non-selective channel of the mitochondrial inner membrane, named the “mitochondrial megachannel”, “multiconductance channel” or mPTP [12, 20]. The mPTP is a ~1.5 nS conductance channel that is activated by voltage and Ca^2+^ and inhibited by CsA. If c-ring forms the largest conductance channel of the mitochondrial inner membrane, then its depletion should eliminate the large-conductance, Ca^2+^ and CsA-sensitive activity of the inner membrane. We set out to determine whether the decrease of free c-subunit and the overall reduction of ATP synthase level would result in significant changes in channel activity.

We used mitoplasts (mitochondrial inner membrane preparations lacking the outer membrane) isolated from WT and c-subunit KD ESCs for single-channel analysis. Patch-clamp recordings of WT mitoplasts demonstrated similar channel activity to that described previously for mPTP [3, 12], with the peak conductance of 1.3 nS and average conductance of 300 pS in the presence of Ca^2+^ and sensitivity to inhibition by CsA (Fig. 4H, J, L, Supplementary Fig. S4B). KD mitoplasts demonstrated channel activity of smaller conductance (~ 300 pS) that was not sensitive to Ca^2+^ and CsA (Fig. 4I, K, L). The voltage ramp recordings showed that channel activity of WT mitochondria not pre-exposed to Ca^2+^ was activated by Ca^2+^ addition and enhanced at negative potentials (Fig. 4J). In contrast, the activity of the KD mitochondria had a reduced sensitivity to voltage and Ca^2+^ (Fig. 4K, L). Our data suggest that c-subunit depletion eliminates the major contributor to Ca^2+^ and CsA-sensitive large conductance channel (mPTP-like) activity of the inner membrane. The smaller, ~300 pS conductance activity found in KD mitoplasts may be related to ANT (Supplementary Fig. S4C). Our data are in agreement with the recent patch-clamp analysis of mitoplasts isolated from the complete c-subunit knockout HAP1-A12 cells first studied in [35]. A small (~300 pS) channel was still found to be present in the c-subunit knockout mitoplasts [36]. This activity was sensitive to bongkrekic acid, suggesting the role of ANT in at least some of the 300 pS conductance activity. These findings suggest that the c-ring is the largest conductance channel of the inner mitochondrial membrane contributing to Ca^2+^ and CsA-sensitive mPT, however ANT also may contribute to lower conductance single ion channel activity of mitochondrial membranes.

## Discussion

Here we show that the highly purified human ATP synthase c-ring forms a large multi-conductance voltage-gated ion channel that undergoes inactivation by purified F_1_ application. We also find that a series of events occurs in primary hippocampal neurons during glutamate excitotoxic treatment (intracellular Ca^2+^ elevation): A Ca^2+^-induced conformational change in ATP synthase leads to dissociation of ATP synthase dimers, dissociation of ATP synthase F_1_ subcomplex from F_O_, and a change in the F_1_/c stoichiometric ratio, predisposing neurons to cell death. Proteolysis of F_1_ occurs over time, but is not required for cell death. Inhibition of proteolysis prevents F_1_ loss, but does not prevent cell death associated with F_1_ detachment suggesting that F_1_ dissociation is non-reversible in severe pathological conditions leading to cell death. These findings highlight the importance of F_1_/c subunit stoichiometry in regulating ACLC activity and confirm the role of F_1_ in forming a gate of the channel. Interestingly, an age-dependent decrease of F_1_ content with respect to that observed for Fo has been reported for rat brain and heart mitochondria [37]. Reduced expression levels of F_1_ subunits and age-dependent dissociation of ATP synthase dimers have also been reported for synaptic mitochondria of the Alzheimer’s disease 5xFAD model mouse [38].

As a more direct experiment of free (uncomplexed with F_1_) c-ring-induced cell death, we performed c-subunit overexpression in neurons undergoing glutamate excitotoxicity and HEK 293 cells undergoing oxidant-induced death. Our data show that c-subunit overexpression aggravates glutamate-induced cell death in primary hippocampal neurons (Fig. 3E–G) and in H_2_O_2_-induced cell death in HEK 293 cells (Fig. 3H, I), while c-subunit KD by shRNA in neurons undergoing glutamate excitotoxicity prevents death. As we show in Fig. 3J, c-subunit is only partially assembled in the ATP synthase complex upon its overexpression in HEK293 cells and it is therefore also present in the mitochondrial membranes as a free c-ring implying that c-ring uncomplexed with F_1_ places cells at risk for death. We suggest that in cells with a reduced F_1_/c-ring ratio, the increased amount of free c-ring increases the probability of ACLC opening in a voltage dependent manner in response to stimuli that induce mPT, whereas without mPT stimuli, there is no evoked inner membrane depolarization and therefore a low probability of voltage dependent mPTP opening (Fig. 3E, G).

Our data implicate c-subunit induced membrane conductance changes in the onset of cell death. It, therefore, seemed prudent to independently assess the recent findings suggesting that ATP synthase c-subunit is not required for mPT [35]. We created c-subunit CRISPR KD embryonic stem cells (with 85% depleted c-subunit) to independently characterize the morphology and single-channel activity of KD mitochondria. The BNP analysis of KD mitochondria suggests that there is a low amount of ATP synthase monomer and no dimers or free c-ring present. Electron micrographs demonstrate the severe change in normal cristae morphology and matrix swelling of the KD cells. These results were not surprising since the ATP synthase dimers are known to be crucial for normal cristae morphology [31, 32]. Although in WT ESCs, Ca^2+^ treatment caused typical changes in electron lucency and spectrophotometric changes in isolated mitochondria, no responses were found in the KD ESCs or isolated mitochondria after Ca^2+^ addition. These findings suggest that c-subunit KD cells have a different mitochondrial phenotype: There are baseline disturbances in mitochondrial inner membrane morphology due to the lack of ATP synthase dimers and in osmotic regulation of matrix volume, allowing us to suggest that ATP synthase and perhaps its c-subunit channel are required for matrix osmotic regulation. It is therefore likely that loss of ATP synthase is associated with disruption of ion gradients across the inner mitochondrial membrane. Such gradients are essential for the normal mitochondrial K^+^ cycle, which consists mainly of influx and efflux pathways for K^+^, H^+^ and associated anions between the matrix and the intermembrane space. The K^+^ cycle is known to be mediated by passive diffusion (“K^+^ leak”), mitochondrial K^+^/H^+^ antiporter and mitochondrial ATP-sensitive K + channel (mitoK_ATP_) [39, 40]. The mitochondrial K^+^ cycle has been reported to be crucial for mitochondrial matrix volume homeostasis, preventing excess matrix swelling and maintaining the structural integrity of mitochondria [39, 40]. We also show that, despite the baseline osmotic changes, CypD and Ca^2+^ induced mitochondrial swelling do not occur, suggesting absence of evoked mPTP opening in c-subunit KD cells.

Our patch-clamp recordings also reveal that c-subunit KD eliminates the large conductance, Ca^2+^ and CsA-sensitive mPTP-like openings present in WT. Our data suggest that although the ACLC may not be the only contributor to mPT under conditions of Ca^2+^ overload and cell stress, it is most likely the largest conductance contributor to CypD-regulated mPT.

Nevertheless, the HAP1-A12 cells with complete knockout (KO) of the ATP synthase c-subunit were recently shown to have no change in the sensitivity of the mPT to calcium during calcium retention capacity (CRC) experiments, allowing the authors to conclude that c-subunit is not required for mPT [35]. The CRC experiments, however, only indicate a loss of membrane potential and not mPT-induced swelling. In fact, when swelling was studied in c-subunit knockout HAP1-A12 cells, there was a marked loss of normal mPTP-induced swelling behavior [41].

The role of ANT in mPTP formation was studied in *Ant*-triple-null (*Ant1*, *Ant2*, *Ant4*) and quadruple-null mice (*Ant1*, *Ant2*, *Ant4, Ppif*). The mPTP opening was inhibited in mouse embryonic fibroblasts (MEFs) of *Ant*-triple-null mice but not in liver cells, which required also the genetic deletion of *Ppif* (CypD), suggesting that ANT may constitute a pore of mPT in MEFs but not in liver cells [21]. Another interpretation of these findings is that ANT is an important regulator of mPTP, rather than a pore-forming component, possibly by maintaining the physiological balance of adenine nucleotides between the matrix and cytosol. If complete deletion of ANT leads to accumulation of the ATP/ADP pool in the matrix, then this will inhibit mPTP channel activity, which would be observed as an inability to depolarize the inner membrane in a calcium-dependent manner, coupled to reduced channel activity as it has been reported for ANT depleted cells [21]. Our findings do not rule out the possibility of the existence of more than one pore of mPT in mitochondrial inner membranes. Nevertheless, our data suggest that the c-ring is the largest conductance channel of the inner mitochondrial membrane contributing to Ca^2+^ and CsA-sensitive mPT.

Another reason for the controversy surrounding the idea of c-ring as the pore of mPT is the hydrophobic nature of the c-ring pore-lining residues. Different types of densities were found to occupy the c-ring lumen in recent structures of ATP synthases from bacteria to eukaryotes. In some studies, the c-ring lumen has been reported to be filled with detergents, lipids or quinones [42–46], which are predicted to preclude ion conductance. c-ring has also been shown to be filled with a 40 amino acid-long alpha-helical protein instead of lipids in the cryo-EM structure of porcine ATP synthase tetramer [14]. 6.8PL proteolipid subunit of ATP synthase was assigned to this density although the most recent high-resolution structure of dimeric ATP synthase reports a different location for 6.8PL and suggests that the c-ring lumen is occupied by phospholipids, as suggested previously [46]. Interestingly, in both of these structures, the C-terminal tail of subunit e interacts with the densities occupying the c-ring [11, 14, 46]. A similar curved region of a presumed F_O_ protein interacting with the detergent micelles of the c-ring cavity was reported earlier for the bovine ATP synthase [1]. Based on this structure the “death finger” model was suggested [47], which proposed that the curved region of F_O_ (recently identified as subunit e [14]) may play an important role in removing the lipid plug from the c-ring.

**Fig. 5:**
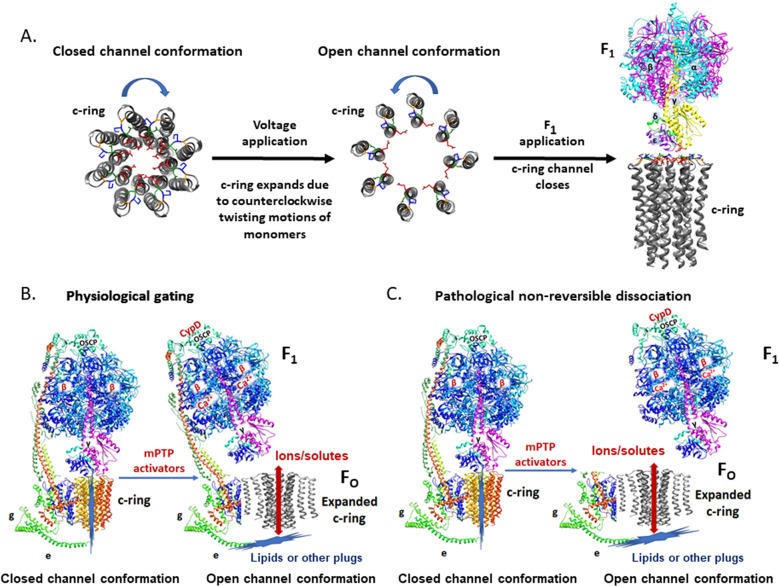
Proposed “bent-pull-twist” model of ACLC gating in physiological and pathological conditions. **A** Illustration of the c-ring diameter changes during the electrophysiology recordings, upon the application of voltage and F_1_. We suggest that F_1_ application during c-ring recordings inactivates the channel due to the specific interactions between F_1_ and c-ring that induce twisting motions in c-subunit monomers in a clockwise direction, to stabilize the ring, reduce the pore diameter and close the channel. **B** Reversible brief openings of ACLC in physiological conditions. **C** Non-reversible dissociation of F_1_ from F_O_ occurs during long-lasting openings of c-ring channel in severe pathology. For simplicity only ATP synthase monomer is shown. ATP synthase subunits are drawn as ribbon representations (modified PDB ID code: 6J5I) [14]. In **B** and **C**, red arrows indicate the path of ion flow through the channel. Closed and open conformations of the channel are noted.

Now, based on the recent structures of ATP synthase, our electrophysiology findings and cell death studies in neurons, we suggest a new “bent-pull-twist” model for ACLC gating which highlights the importance of F_1_ gating. We suggest that during the mPT initiation step, mPTP modulators bind directly to different ATP synthase subunits: CypD binds to OSCP subunit [23, 48, 49] and Ca^2+^ binds to β subunit [6, 50] (Fig. 5). OSCP connects F_O_ with F_1_ through the peripheral stalk and is an important site of modulation of ATP synthase leak channel activity due to its interaction with different endogenous and pharmacological inducers of mPTP [51, 52]. We suggest that these binding steps induce conformational changes in the ATP synthase peripheral stalk subunits, which then modify interactions of c-subunit with the other F_O_ subunits. The subsequent conformational changes may then pull away F_1_ from the mouth of the c-ring pore to free the channel from the side facing the matrix. These conformational changes may also pull out or displace the lipid or other “plugs” from the c-ring lumen to free the channel from the side facing the intermembrane space. We suggest that the c-ring lumen expands during these concurrent events to facilitate channel opening (Fig. 5). We have reported earlier the expansion in the c-ring diameter during Ca^2+^ induced mPT [3] and it is supported here by the behavior of continuously increasing channel conductance of the c-ring (Fig. 1D). According to cryo-EM structures of mammalian c-ring, the eight monomers are twisted clockwise [14]. Here we suggest that this corresponds to the narrow pore, closed channel conformation of ATP synthase. We speculate that during the above-mentioned conformational changes, c-subunit monomers twist counterclockwise, which widens the pore and induces the opening of the channel (Fig. 5A). F_1_ application during c-subunit recordings inactivates the channel since the specific interactions between F_1_ and c-ring [53, 54] induce twisting motions in c-subunit monomers in a clockwise direction, stabilizing the ring, reducing the pore diameter and closing the channel (Fig. 5A). The model in Fig. 5B represents the reversible, brief openings of the ACLC, the type of openings that most likely occur under physiological or sub-lethal conditions. We suggest that in severe pathology such as brain ischemia represented here by an extended period of glutamate toxicity, non-reversible dissociation of F_1_ from F_O_ occurs following long-lasting openings of ACLC (Fig. 5C). These events are accompanied by inability to repolarize the inner membrane. This marks the point of no return because marked matrix swelling as a result of prolonged pore opening triggers outer membrane rupture, release of cytochrome c and activation of downstream cell death pathways.

Our proposed model highlights the crucial role of c-ring as the main pore-forming component of mPTP [3, 4], the role of F_1_ as a gate of ACLC and the auxiliary roles of the other ATP synthase subunits, OSCP, e and g [5, 9, 10, 55]. Our findings and model suggest that the c-subunit channel is activated by voltage and gated by F_1_ from the side of the matrix. ACLC may possibly have a second gate (lipids or other plugs), from the side of the intermembrane space as suggested before [11, 47]. These plugs may be partially displaced or completely removed by voltage application (in in vitro experiments) or due to membrane depolarization (in in vivo) (Fig. 5). Further electrophysiological studies are needed to fully characterize the gating mechanism of ACLC.

Similarly to our current studies on excitotoxicity, we reported previously that levels of the ATP synthase F_1_ β subunit are low in aged DJ-1 deficient, Parkinson’s Disease (PD) mouse model brain and in patient-derived cell lines, while the c-subunit levels are normal, suggesting that mutant DJ-1-induced degenerative disease is associated with a reduction in F_1_/c ratio with resultant ACLC activation [56]. Likewise, we have recently reported a similar reduction in F_1_/c ratio in Fmr1 deficient mouse neurons [26]. Fmr1 encodes the Fragile X mental retardation protein (FMRP) which we find regulates the closure of the ACLC by increasing the translation rate of F_1_ β subunit, thus increasing the F_1_/c ratio, enhancing ATP synthesis efficiency, resulting in synaptic growth [26, 57].

Reversible disassembly is a well-known occurrence of the vacuolar ATPase (V-ATP-ase) which has a remarkable similarity with ATP synthase. Interestingly, it was shown that c-ring isolated from yeast V-ATPase forms a large unitary conductance channel [58].

Occlusion of other known ion channels with lipids and a hypothetical gating mechanism, termed “lipid gating” has been reported recently [59]. Another possible mechanism of c-ring conductance was suggested to involve the Ca^2+^/CypD-dependent association of polyhydroxybutyrate-polyphosphate complex with the c-ring [60].

In-depth structural and functional studies of ATP synthase are required to increase understanding of the conformational changes accompanying ACLC opening and to better characterize this mitochondrial “death” channel which is ironically located in the main energy-producing enzyme of the cell.

## Methods

### Purification of c-subunit, F_1_, α_3_β_3_ complex and c-subunit knock-down

The human ORF ATP-synthase c1 (ATP5G1) subunit construct tagged on the C terminus with Myc and DDK (Flag) was used (Origene Technologies). The construct for c-subunit was expressed in HEK 293 cells and purified using the EZ view Red ANTI-FLAG M2 Affinity Gel (Sigma), according to the manufacturer’s protocol.

Endogenous c-subunit knockdown (for ATP5G1 gene) was performed using a plasmid, expressing short hairpin RNAs (shRNA) (Origene Technologies).

The sequence of human ATP synthase c-subunit corresponding to mature protein was cloned into the bacterial pEX-1 expression vector (BlueHeron Biotech). DNA codon optimization strategy was used to increase the expression level of protein in *E. coli* cells (Supplementary Fig. S1C). The protein was tagged with six histidine residues at its C-terminus to facilitate purification (Ni-NTA).

F_1_ ATPase was purified from HEK 293 cell mitochondria according to a previously published protocol [61]. The α_3_β_3_ complex (*G. stearothermophilus*) was purified according to previously published protocols [62].

### Proteoliposome preparation

Proteoliposomes were prepared according to published protocols [3]. Briefly, 50 mg of phosphatidylcholine (Sigma) or phosphatidylcholine, phosphatidylethanolamine and cardiolipin (3:3:1 ratio) (Avanti) mixture was dissolved in 1 mL of chloroform. A thin lipid film was formed on a glass surface by evaporating the chloroform. Liposomes were formed by the reconstitution of the lipid in rehydration buffer containing 250 mM KCl, 5 mM HEPES, and 0.1 mM EDTA. Then, 20 μg of recombinant c-subunit protein was added to 100 μL of the liposome mixture (∼2 mg of lipids, final), and the samples were vortexed twice. For recording, proteoliposomes were dehydrated in a recording chamber and rehydrated in a recording solution (120 mM KCl, 8 mM NaCl, 0.5 mM EGTA, 10 mM HEPES (pH 7.3)) in the presence of SM2 Bio-Beads (Bio-rad) to remove the detergent (2 h, room temperature or overnight at 4 °C). The presence of the recombinant c-subunit in the liposomes was confirmed by immunohistochemistry using anti-Myc mouse monoclonal (AlexaFluor 488 conjugate) antibody (Cell Signaling), according to published methods [3].

### Generation of c-subunit knockdown cells via CRISPR/Cas9

Two guide RNA target sites specifically designed for each ATP synthase c-subunit gene (ATP5G1, 2, 3) for mouse embryonic stem cells (ESCs) were selected using the CRISPOR website (https://tefor.net/portfolio/crispor). The selected sequences were: ATP5g1T:CACCGCACCCTTTTCTGTCTCTGGC, ATP5g1B:AAACGCCAGAGACAGAAAAGGGTGC; ATP5g2T: CACCGTGCTTCAGAGAAGGGTTCCT, ATP5g2B: AAACAGGAACCCTTCTCTCTGAAGCAC; ATP5g3T: CACCGAACAGCTGCTGCTTCAGTGA; ATP5g3B: AAACTCACTGAAGCAGCAGCTGTTC.

Oligos for these sequences were annealed and ligated into the pX330 plasmid (Addgene) that was cut with the BbsI restriction enzyme. Cas9/sgRNA expressing plasmids were electroporated into mouse ES cells via standard techniques (Manipulating the Mouse Embryo: A Laboratory Manual, 3^rd^ ed. (Nagy et al., eds.) 2003: Cold Spring Harbor Laboratory Press, Cold Spring Harbor, NY). The efficiency of knockdown was verified by Western blot analysis using antibodies against the c-subunit (Abcam, Cat. No. 181243).

### Mitochondrial isolation from primary neuronal cultures, HEK 293 cells, and mouse ES cells

Mitochondria from primary hippocampal and cortical neurons, HEK 293 and mouse ES cells were isolated using the Qproteome Mitochondria isolation kit (Qiagen, Cat. No. 37612). In brief, cells were transferred to ice-cold isolation buffer, supplemented with 1x Halt protease inhibitor. Cells were minced, homogenized with a Dounce homogenizer, and centrifuged at 1000 × *g* to pellet nuclei, cell debris, and unbroken cells. The supernatant was centrifuged at high-speed (6000 × *g* for 15 min at 4 °C); the pellet containing mitochondria was washed in isolation buffer and pelleted by centrifugation at 6000 × *g*. Feeder cells were depleted before mitochondria isolation from mouse ES cells. Protein concentration was determined by the BCA method using BSA as a standard. Isolated mitochondria were used immediately or stored at −80 °C until use.

### Swelling assay

To initiate mitochondrial swelling by Ca^2+^ uptake, freshly isolated mitochondria were suspended in 120 mM KCl, 25 mM sucrose, 5 mM KH2PO4, 0.1 mM EGTA, 20 MOPS (pH 7.2) in the presence of 5 mM malate and 5 mM pyruvate as substrates, in the presence or absence of CsA (1 uM). A membrane-permeabilizing nonspecific agent, alamethicin (10 μM) was used to determine if mitochondria were capable of swelling. The swelling was recorded as the decrease of the density of the mitochondrial matrix at 540 nm with a UV/Vis spectrophotometer after adding 0.5 mM Ca^2+^ into the medium.

### Mitochondrial oxygen consumption and membrane potential measurement in isolated mitochondria

Mitochondria (0.2 mg) isolated from mouse embryonic stem cells were added to 1 ml of respiration buffer [200 mM sucrose, 25 mM KCl, 2 mM K_2_HPO_4_, 5 mM HEPES-KOH (pH 7.2), 5 mM MgCl_2_, 0.2 mg/mL BSA]. Oxygen consumption and mitochondrial membrane potential were measured simultaneously [63]. Oxygen consumption was measured using a Clark‐type electrode (Oxytherm, Hansatech, UK) with Complex II‐dependent substrate (10 mM succinate in the presence of 5 µM rotenone) at 32 °C. Membrane potential was determined using an ion-sensitive electrode selective for the lipophilic cation, tetraphenylphosphonium (TPP+) (World Precision Instruments, Sarasota, FL), and calculated using the Nernst equation as previously described [57]. ADP (200 µM) was added to initiate ADP-dependent respiration.

### Electrophysiology

The patch-clamp recordings of mitoplasts and ATP synthase c-ring reconstituted liposomes were performed by forming a giga-ohm seal in intracellular solution (10 mM Hepes, pH 7.3, 120 mM KCl, 8 mM NaCl, 0.5 mM EGTA,) using an Axopatch 200B amplifier (Axon Instruments) at room temperature (22–25 °C). Recording electrodes were pulled from borosilicate glass capillaries (WPI) with a final resistance in the range of ~50 MΩ. Signals were filtered at 5 kHz using the amplifier circuitry.

Planar lipid bilayer recordings were performed in intracellular solution by using the α-L-phosphatidylcholine (Sigma) or α-L-phosphatidylcholine and cardiolipin (Avanti) in 3:1 ratio for forming the bilayer membrane. ePatch amplifier (Elements) was used for lipid bilayer recordings. ATP (1 mM, final concentration), bongkrekic acid (BA, 5 µM), purified F_1_ (5 µg, final concentration) and α_3_β_3_ complex (5 µg) were added into the bath during the recordings without perfusion.

pCLAMP-10 software was used for electrophysiology data acquisition and analysis (Molecular devices). All current measurements were adjusted for the holding voltage assuming a linear current-voltage relationship: The resulting conductances are expressed in pS according to the equation *G* = *I/V* where *G* is conductance in pS, *V* is the membrane holding voltage in mV, and *I* is the peak membrane current in pA after subtraction of the baseline electrode leak current. Group data were quantified in terms of conductance and probability of channel opening, where NPo is the number of open channels (“level” in pCLAMP) times the probability of channel opening at each level. All population data were expressed as mean ± SEM.

### Primary cultures of rat hippocampal neurons

Primary rat hippocampal neurons were prepared from rat feti (Sprague-Dawley, day 18 of gestation; Harlan, Indianapolis, IN) as described previously [3] with modifications specific for this study. After isolation of hippocampi from prenatal brains, neurons were dissociated and seeded (0.2 ×106 cells/ 35 mm plate) onto plates containing medium with 5% FBS. After 2 h incubation, cells were maintained in Neurobasal medium supplemented with B-27, glutamine, and antibiotics (Invitrogen GIBCO life technologies, Carlsbad, CA). Neurons were grown at 37 °C in 5% CO_2_ and 20% O_2_ in a humidified incubator and treated at DIV 20–21. Glutamate treatment: glutamate (monosodium glutamate, 20 μM, final concentration), (Sigma-Aldrich, St. Louis, MO) was freshly made in sterile PBS as an aqueous solution then added to the cell culture medium as described in relevant figure legends. The vehicle group for glutamate experiments is treated with sterile PBS. Cyclosporine A (CsA) treatment: CsA (Cell signaling) was prepared in sterile ethanol and added to the cell culture medium (100 nM, final concentration). Proteasome inhibitor (PI) treatment: MG-132 (ApexBio, Houston, TX) was prepared in DMSO and added to the cell culture medium (0.1 μM, final concentration).

### Viability assay

Lactate dehydrogenase assay: The level of cytotoxicity in primary hippocampal neurons was assayed by measuring leakage of LDH using an in vitro toxicology assay kit (Sigma- Aldrich) as previously described [64]. In brief, data were calculated by finding the activity of LDH leaked into the medium by damaged cells/total LDH activity in the culture (cells plus medium). The culture media and lysed cells were collected after the treatment of neurons as described in the relevant figure legends. The LDH assay mixture was made according to the manufacturer’s protocol and added to each sample. After 20 min incubation, the reaction was terminated by adding 1 N HCl. LDH activity was spectrophotometrically measured with a VICTOR multilabel reader (PerkinElmer, Waltham, MA, USA) with absorbance set at 490 nm.

### Cell death assay

Annexin V and Propidium iodide (PID): Apoptotic or total dead cells were stained with Annexin V or PID, respectively, as previously described [27]. After treatment of neurons expressing GFP, GFP plus c-subunit shRNA or GFP plus human c-subunit constructs with glutamate for 18 h, 5 μl/1 ml Annexin V or 0.5 μM PID (Thermo Fisher Scientific, Waltham, MA) was added into the culture medium for 30 min at 37 °C in the dark. Micrographs were taken using a Zeiss Axiovert A1 microscope (Zeiss, OberKDchen, Germany) using a consistent exposure time. The number of PID-positive neurons or Annexin V fluorescence densitometry per cell was analyzed using AxioVision 4.9. The H_2_O_2_-induced cell death in HEK 293 cells was measured with PID after transfected the cells with constructs expressing GFP or GFP and human c-subunit constructs. Cell death was measured 24 h after a 30 min. exposure to 1 mM H_2_O_2_.

### Measurement of mitochondrial potential in primary hippocampal neurons

Mitochondrial membrane potential (Δψ) was measured using the fluorescent lipophilic cationic dye tetramethylrhodamine methyl ester (TMRM, Invitrogen, ThermoFisher, Waltham, MA), which accumulates within mitochondria in a potential-dependent manner [64, 65]. Primary hippocampal neurons were treated with glutamate (20 μM) for 6 h and TMRM intensity was measured to assess the mitochondrial membrane potential. Primary hippocampal neurons were stained with 5 nM TMRM for 30 min at 37 °C in the dark. Cells were pre-incubated with CsA (100 nM, 30 min) before TMRM was added. Images were taken using a Zeiss Axiovert A1 microscope and TMRM fluorescence densitometry was analyzed using AxioVision 4.9.

### Immunocytochemistry

Primary hippocampal neurons were transfected with Mitochondria-GFP (Invitrogen) at DIV14, and fixed in 10% buffered formalin at DIV 20–22. Then, samples were incubated with anti-Flag (1:500 dilution, Origene, Rockville, MD) overnight at 4 °C. Cells were washed and incubated with Alexa-568 antibody (1:200 dilution, Invitrogen, Molecular Probes, Carlsbad, CA) for 1 h at room temperature and mounted on glass slides. Images were taken with Zeiss LSM 710 confocal scanning microscope (Zeiss, OberKDchen, Germany) and processed using ZEN software (Carl Zeiss Microscopy GmbH, Jena, Germany).

### Electron microscopy

Mouse ES cells were fixed in 2.5% glutaraldehyde in 0.1 M sodium cacodylate buffer, pH 7.4 for 1 h. Buffer rinsed cells were scraped in 1% gelatin and spun down in 2% agar. Chilled blocks were trimmed and postfixed in 1% osmium tetroxide for 1 h. The samples were rinsed three times in sodium cacodylate rinse buffer and postfixed in 1% osmium tetroxide for 1 h. Samples were then rinsed and stained in aqueous 2% uranyl acetate for 1 h followed by rinsing, dehydrating in an ethanol series, infiltrated with Embed 812 (Electron Microscopy Sciences) resin, and then baked overnight at 60 °C. Hardened blocks were cut using a Leica UltraCut UC7. Sections (60 nm) were collected in formvar/carbon-coated nickel grids and contrast stained with 2% uranyl acetate and lead citrate. They were viewed using a FEI Tencai Biotwin TEM at 80 Kv. Images were taken on a Morada CCD using iTEM (Olympus) software.

Purified human ATP synthase c-ring was stained with 2% uranyl acetate. Negative- stain electron microscopy images were taken by using FEI Tecnai TF20 microscope (FEG, 200 kV) (Yale Center for Cellular and Molecular Imaging).

### Blue and clear native page electrophoresis

For Blue Native Page (BNP) electrophoresis protein complexes from 20 µg of mitochondria (per lane) were separated on a Bis-Tris 3–12% Native gels. Samples were solubilized on ice for 20 min with 4 µg digitonin/µg protein or 2.5 ug n-dodecyl β-D-maltoside (DDM)/ug protein. After separation, the protein complexes were wet-transferred onto a polyvinylidene fluoride (PVDF) membrane, which was then probed with anti-ATP5A, ATP5B and anti- ATP5G1,2,3 antibodies for ATP synthase alpha-, beta- and c-subunits. An aliquot of each sample prepared for BNP electrophoresis was used to verify protein loading of these blots by detection of complex IV, a protein of the inner mitochondrial membrane. Clear Native Page electrophoresis was performed according to the published protocol [66].

### Antibodies

Antibodies used in all experiments were obtained from commercial sources.

### Statistical analysis

Data in graphs are shown as mean ± SEM. For comparisons involving two groups, paired or unpaired Student *t* tests (2-tailed) were used, and exact *P* values are provided in the figure legends. For multiple comparisons, Prism (V6.02; GraphPad) was used to calculate the normality of data using the D’Agostino–Pearson omnibus or the Kolmogorov–Smirnov normality tests. Parametric data were analyzed using standard or repeated-measures ANOVA with Dunnett’s or Tukey’s multiple comparison test whereas nonparametric data were analyzed using Kruskal–Wallis with Dunn’s multiple comparison test and multiplicity adjusted (“exact”) *P* values are reported. In some cases, data were normalized to control data before analysis. Unless noted, other comparisons were not significant.

## Supplementary information

Figure S1

Figure S2

Figuer S3

Figure S4

Supplementary Figure Legends

Original data

## Data Availability

All constructs will be made available to the scientific community upon request.
