# Supplementary figures and images for: Mitochondrial ATP synthase c-subunit leak channel triggers cell death upon loss of its F1 subcomplex
Source: Cell Death Differ. 2022 Mar 23;29(9):1874–87. doi: 10.1038/s41418-022-00972-7 (PMC9433415; doi:10.1038/s41418-022-00972-7)

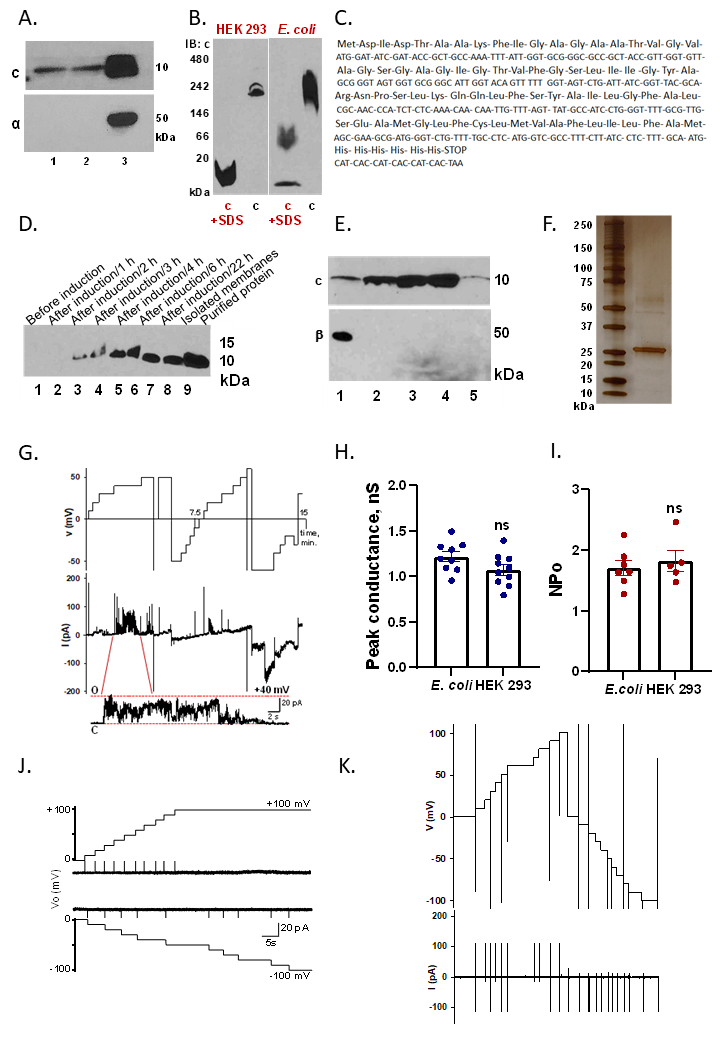

Supplement: Supplementary file 1 — Figure S1 [file 41418_2022_972_MOESM1_ESM.png]

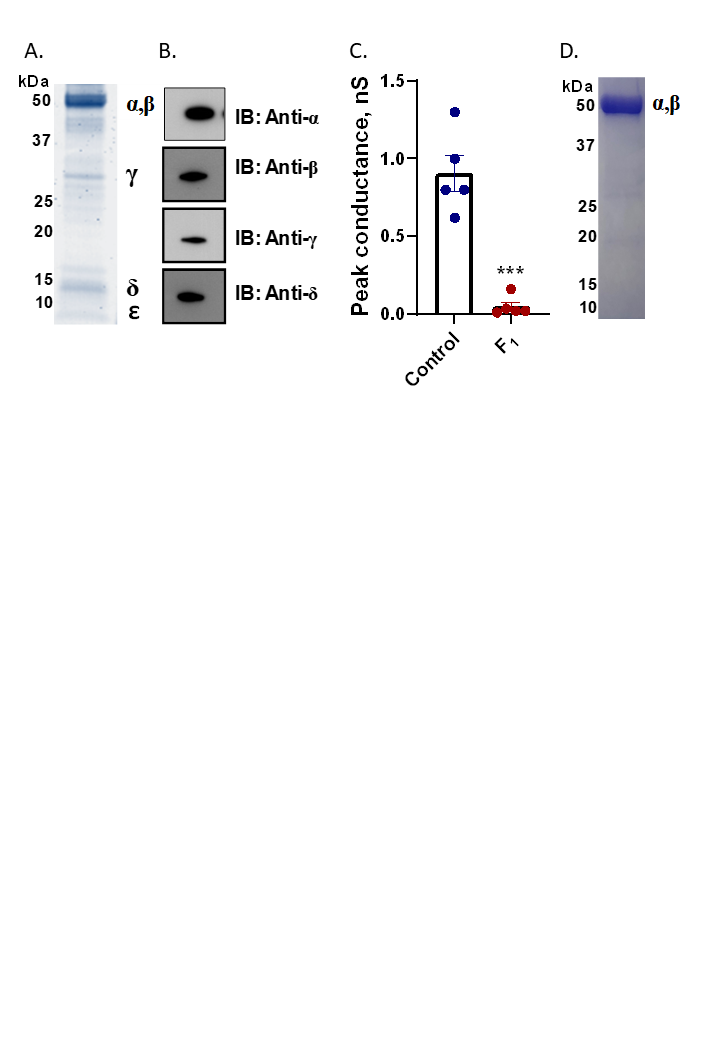

Supplement: Supplementary file 2 — Figure S2 [file 41418_2022_972_MOESM2_ESM.png]

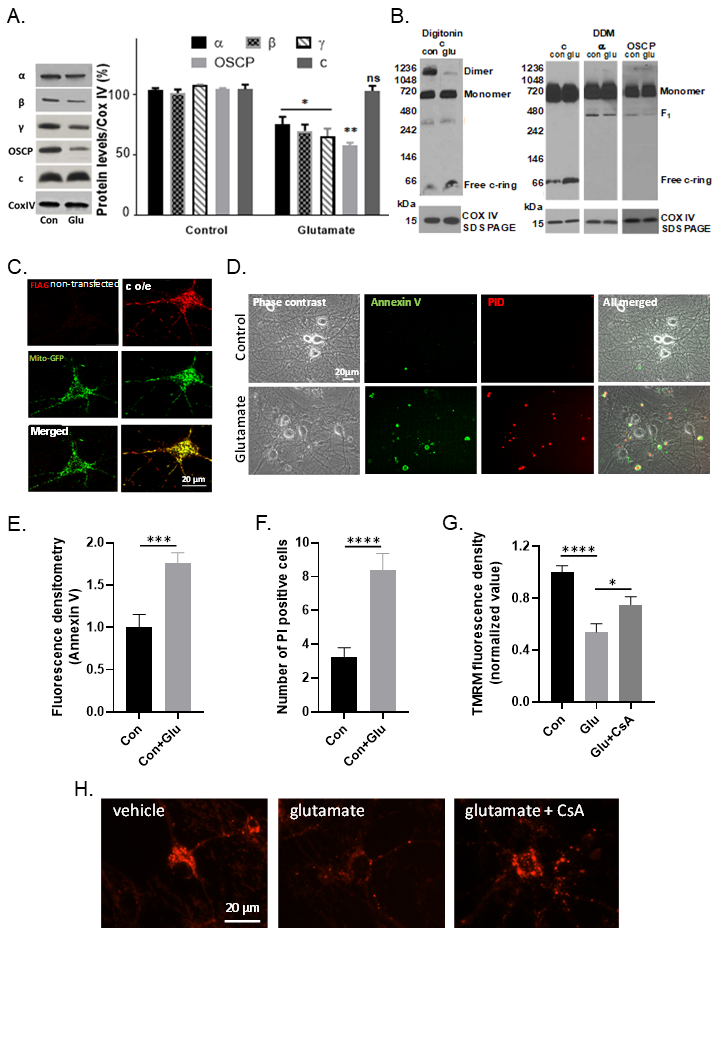

Supplement: Supplementary file 3 — Figuer S3 [file 41418_2022_972_MOESM3_ESM.png]

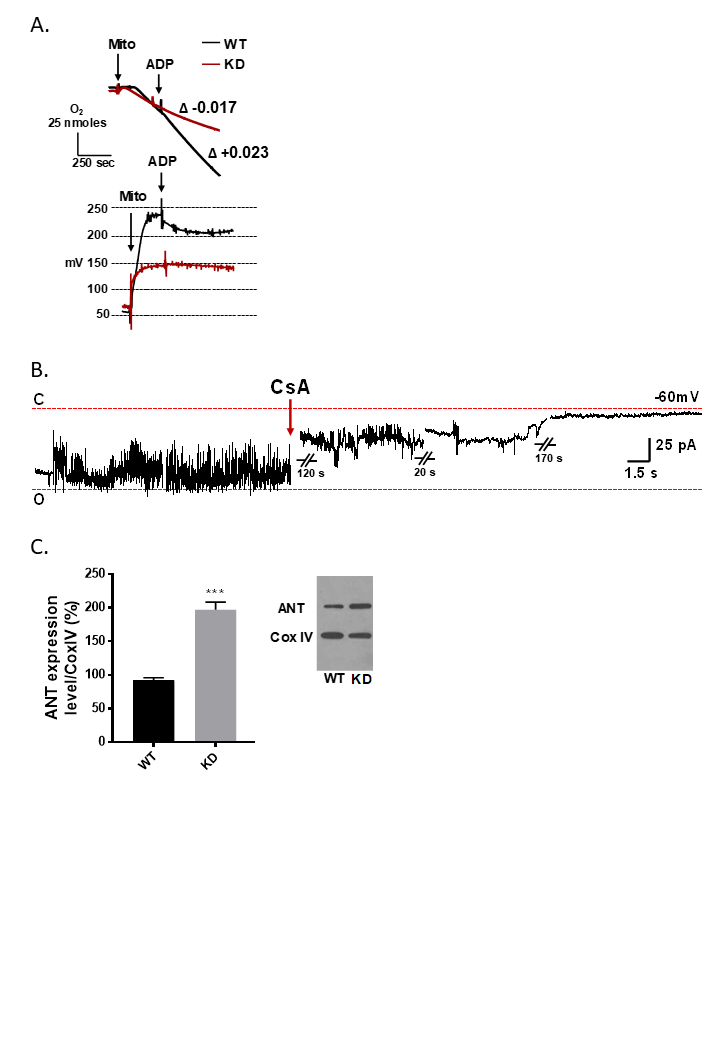

Supplement: Supplementary file 4 — Figure S4 [file 41418_2022_972_MOESM4_ESM.png]
