# Supplementary material for: Mitochondrial ATP synthase c-subunit leak channel triggers cell death upon loss of its F1 subcomplex
Source: Cell Death Differ. 2022 Mar 23;29(9):1874–87. doi: 10.1038/s41418-022-00972-7 (PMC9433415; doi:10.1038/s41418-022-00972-7)
Supplement: Supplementary file 5 — Supplementary Figure Legends [file 41418_2022_972_MOESM5_ESM.docx]

**Supplementary figure legends**

**Figure S1. A.** Immunoblot analysis of c-subunits purified from HEK 293 cells (sample 1, 2, purified c-ring, 3, mitochondrial lysate used as positive control), showing the absence of F_1_ α subunit in purified human c-subunit samples. **B.** Immunoblot analysis of c-subunits purified from HEK 293 cells and *E. coli* after non-denaturing clear Native-PAGE showing that purified proteins preserve their oligomeric state. Purified proteins treated with or without 1% SDS were resolved by clear Native-PAGE and transferred by western blot to a PVDF membrane, which was then probed with the anti-c antibody. Bands with ∼250 kDa size were detected in control (non-denatured) c-subunit samples corresponding to the tetramers of octameric rings, while bands with the sizes of ∼66 kDa and ∼8 kDa corresponding to the intact octameric rings or dissociated c-subunit monomers, respectively, were observed in SDS treated samples (gel is representative of 2 gels). **C.** Amino acid and DNA sequences of human c-subunit used for expression in *E. coli*. DNA codon optimization strategy was used to increase the expression level of human c-subunit in *E. coli*. **D.** Immunoblot analysis of *E. coli* cells overexpressing human c-subunit before and after IPTG induction (samples 1-7). Isolated *E. coli* membranes (sample 8), and purified protein (sample 9) after denaturing SDS-PAGE are shown. The PVDF membrane was probed with the anti-c antibody. **E.** Immunoblot analysis of *E. coli* cell lysate overexpressing human c-subunit (sample 1). The lysate was used as a positive control to probe for *E. coli* F_1_ β subunit; human c-subunit purified by Ni-NTA (samples 2-5) showing the absence of *E. coli* F_1_ β subunit after purification. The PVDF membrane was probed with anti-β (specific to interact with *E.coli* β subunit) and anti-c antibodies. **F.** Purified His-tagged membrane scaffold protein (MSP). The sample was resolved by SDS-PAGE and stained with silver. **G.** Representative continuous lipid bilayer recording of human c-subunit (purified from *E. coli*) and **H.** Group data for peak conductances and **I.** open probability (NPo) of c-ring channels purified from *E. coli* and HEK 293 cells, unpaired t-test. **J.** Representative lipid bilayer recordings at different voltages from -100 mV to + 100 mV of membrane scaffold protein (used as a control; representative of n=5, voltage was changed in 10 mV increments). The small spikes on the current traces represent stepwise changes in voltage. K. “no protein” control experiment recorded at different voltages.

**Figure S2. A.** F_1_ purified from a mitochondrial fraction of HEK 293 cells. The sample was resolved by SDS-PAGE and stained with Coomassie. **B.** Immunoblot analysis of purified F_1_; image from a representative experiment (n=3); the membrane was probed with antibodies for different F_1_ subunits. **C.** Group data for peak conductances in response to the addition of purified F_1_ to c-subunit during planar lipid bilayer experiments**,** n=5, ***P = 0.0008, paired t-test. **D.** α_3_β_3_ purified from *E. coli* resolved by SDS-PAGE and stained with Coomassie. Only one band is seen on the gel due to the similar sizes of α (55 kDa) and β subunits (50 kDa).

**Figure S3. A.** Immunoblot analysis of mitochondria isolated from cortical neurons before and after glutamate treatment (n=3 independent cultures, *P<0.0352, **P<0.005, one-way ANOVA, error bars refer to SEM). Antibodies for different ATP synthase subunits were used as indicated. **B.** Immunoblot analysis of digitonin-solubilized mitochondrial lysate and n-dodecyl β-D-maltoside (DDM)-solubilized mitochondrial lysate from control (con) and glutamate treated (glu) hippocampal neurons, after non-denaturing Blue Native Page. Digitonin as a milder detergent, preserves the dimeric state of ATP synthase. For both types of detergent, F_1_ subunit level (α, OSCP) is reduced in glutamate exposed conditions, while the free c-subunit level is increased. **C.** Immunocytochemistry of cultured hippocampal neurons showing co-localization of overexpressed Flag-tagged c-subunit with mitochondria. Red: Flag; Green: Mito-GFP. **D.** Control and Glutamate-treated primary hippocampal neurons were stained with Annexin V and propidium iodide (PID) to show apoptotic or total dead cells, respectively. Green: Annexin V; Red: PID. After treatment of neurons with Glutamate (20µM) or vehicle (Control) for 18h, Annexin V and PID were added into the culture medium for 30min at 37°C in the dark. **E., F.** Group data of Annexin V and propidium iodide (PID) staining (n=15 micrographs each for Control or Glutamate-treated; the densitometry of Annexin V or the number of PID-positive neurons in 0.05 mm^2^ was calculated). n=3 independent cultures, and 3 independent experiments, ***P<0.001, and ****P<0.0001, one-way ANOVA. **G.** TMRM fluorescence intensity was quantified (n=16 micrographs/group and 3 independent experiments, *P<0.0407, and ****P<0.0001, one-way ANOVA Tukey’s multiple comparisons test) and **H.** TMRM stained neurons were imaged. Primary hippocampal neurons were treated with glutamate (20 μM) and CsA (100nM) for 6 h and TMRM intensity was measured to assess the mitochondrial membrane potential**.** TMRM (5 nM) was added to the cell culture medium.

**Figure S4. A.** ADP-induced oxygen consumption rate and membrane potential of WT and c-subunit CRISPR KD mitochondria isolated from mouse embryonic stem cells (ESCs). The graph shows the oxygen consumption rate before and after the addition of mitochondria and ADP. The values shown on the oxygen consumption curves indicate the change in oxygen consumption after ADP addition. The WT mitochondria increase oxygen consumption after ADP addition, whereas the KD mitochondria have low oxygen consumption before ADP and fail to increase oxygen consumption after ADP addition. The graph is representative of 2 experiments. **B.** Representative patch-clamp recording of WT mitoplast, pre-treated with Ca^2+^ (1mM) at -60 mV holding voltage before and after the addition of CsA (5 uM). **C.** Group data showing the expression level of ANT in WT and c-subunit CRISPR KD mitochondria after western blot analysis with indicated antibodies (n=3, ***P<0.001, unpaired t-test).
