# Supplementary material for: Mitochondrial ATP synthase c-subunit leak channel triggers cell death upon loss of its F1 subcomplex
Source: Cell Death Differ. 2022 Mar 23;29(9):1874–87. doi: 10.1038/s41418-022-00972-7 (PMC9433415; doi:10.1038/s41418-022-00972-7)

Supplementary material

Figure 3 A

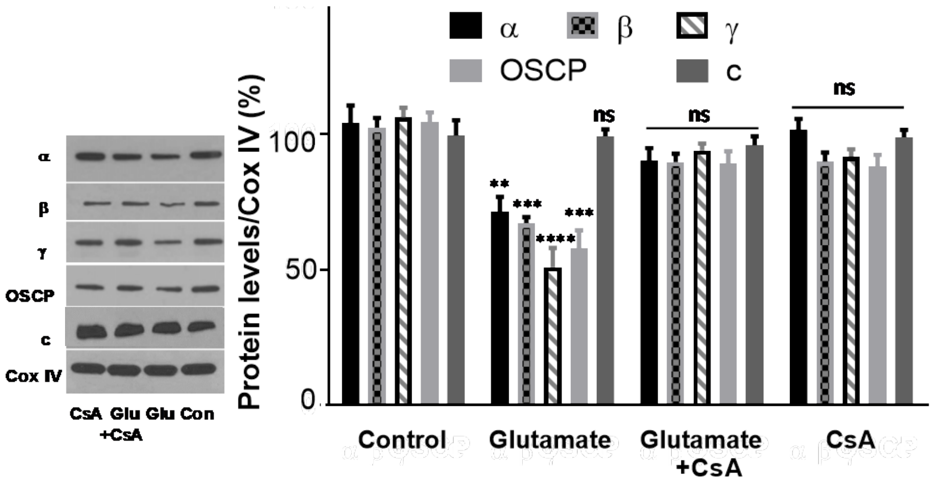
α
β
γ
OSCP
c
Cox IV
CsA Glu Glu Con
+CsA
Protein levels/Cox IV (%)
100
50
0
α β γ
OSCP c
ns
ns
ns
**
***
****
***
Control
Glutamate
Glutamate
+CsA
CsA

Figure 3A

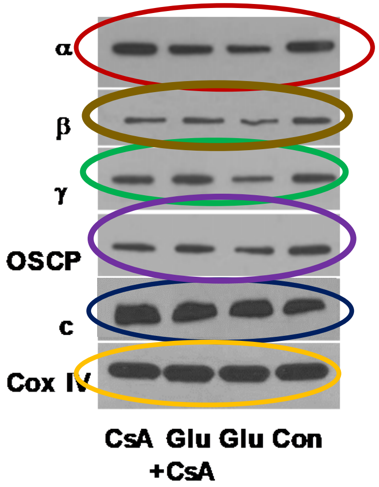
α
β
γ
OSCP
c
Cox IV
CsA Glu Glu Con
+CsA

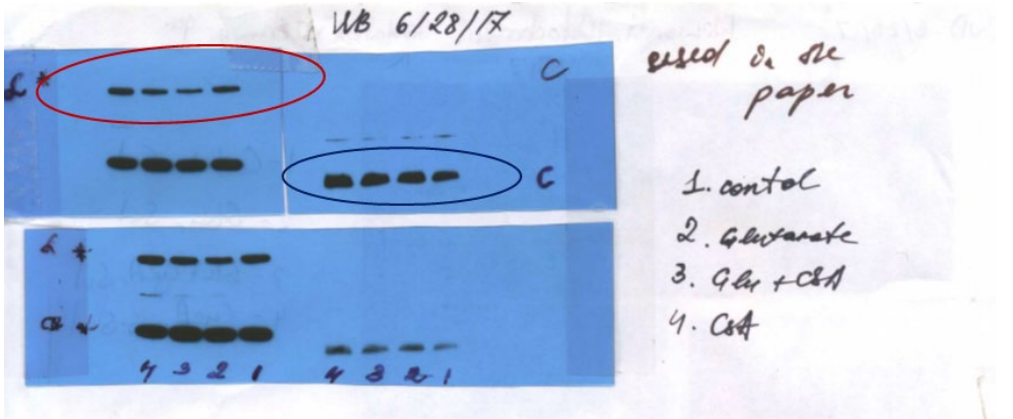
WB 6/28/17
1. control
2. Glutamate
3. Glu + CsA
4. CsA

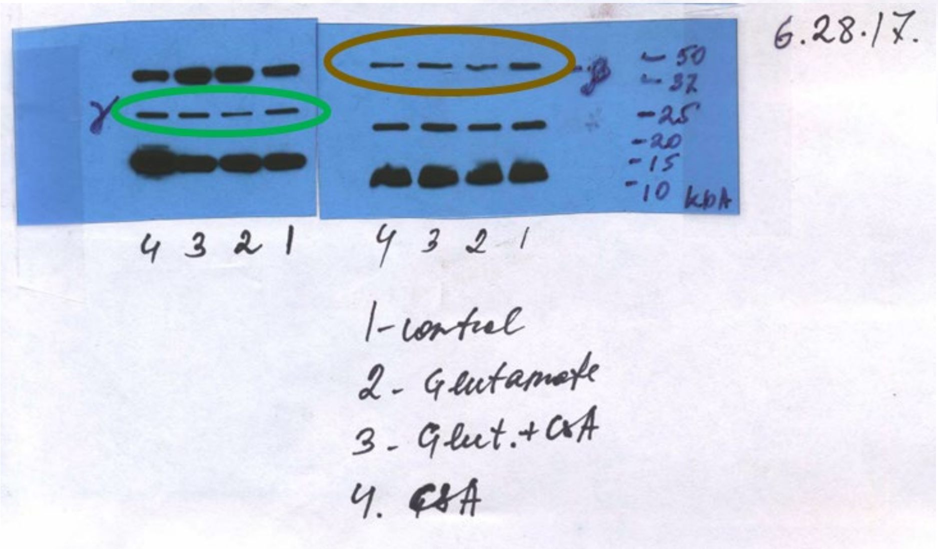
6.28.17.
50
37
25
20
15
10
kDA
4 3 2 1
4 3 2 1
1- control
2- Glutamate
3- Glut.+CsA
4. CsA

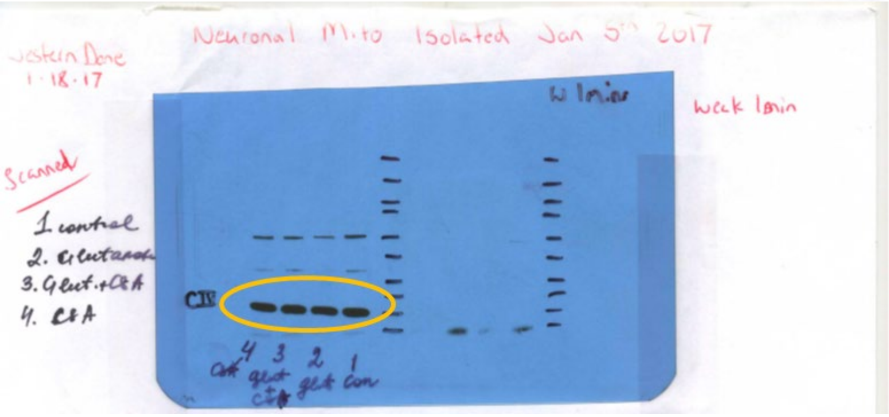
Western Done
1.18.17
Neuronal Mito Isolated Jan 5th 2017
Scanned
1. control
3. Glut.+CsA
4. CsA
weak 1min
CIV

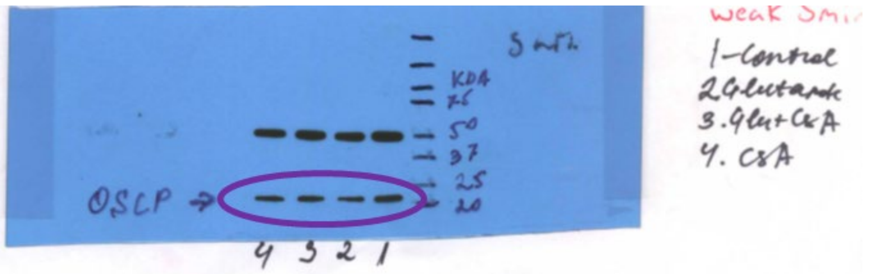
OSCP
1-Control
3.Glu+CsA
4. CsA
4 3 2 1

Figure 3 C

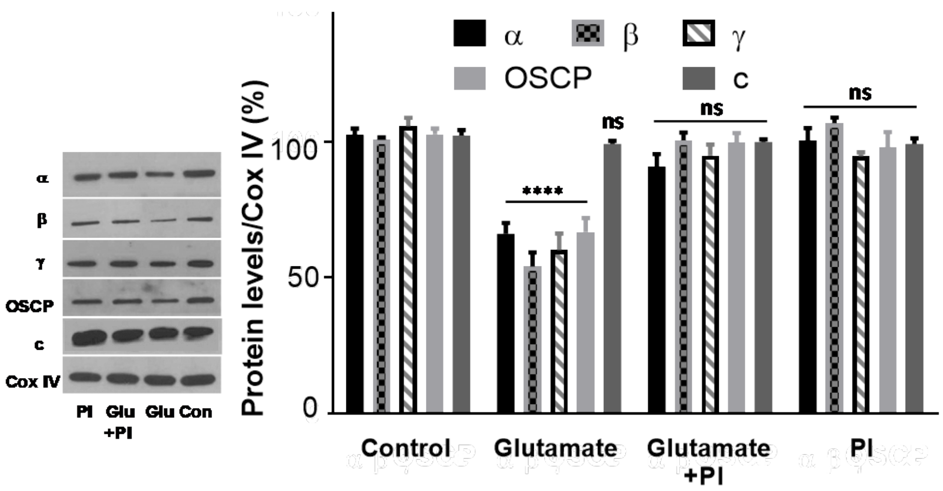
α
β
γ
OSCP
c
Cox IV
PI
Glu +PI
Glu
Con
Protein levels/Cox IV (%)
100
50
0
α
β
γ
OSCP
c
****
ns
ns
ns
Control
Glutamate
Glutamate +PI
PI

Figure 3C

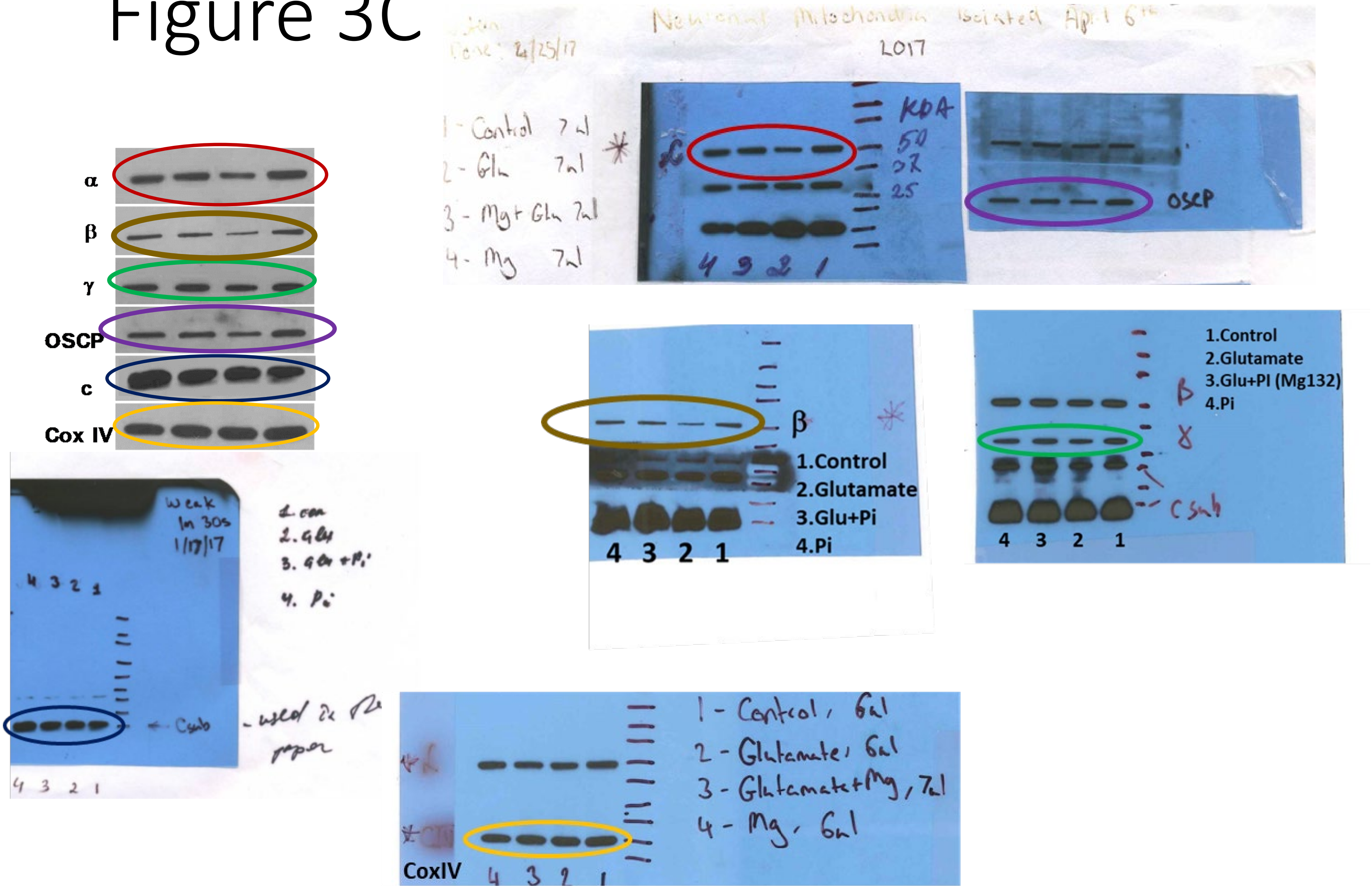

α
β
γ
OSCP
c
Cox IV
New Mitochondria Isolated April 6th 2017
1 - Control 7ul
2 - Glu 7ul
3 - Mg + Glu 7ul
4 - Mg 7ul
KDA
50
37
25
4 3 2 1
OSCP
β
1.Control
2.Glutamate
3.Glu+Pi
4.Pi
4 3 2 1
1.Control
2.Glutamate
3.Glu+PI (Mg132)
4.Pi
4 3 2 1
1. con
2. Glu
3. Glu + Pi
4. Pi
4 3 2 1
Csub
1 - Control, 6ul
2 - Glutamate, 6ul
3 - Glutamate + Mg, 7ul
4 - Mg, 6ul
CoxIV
4 3 2 1

Figure 4A

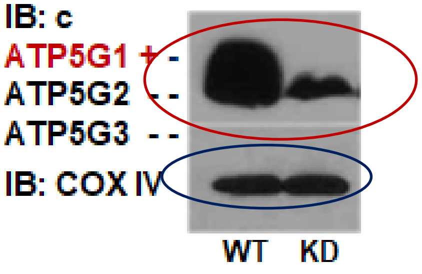
IB: c
ATP5G1 +
ATP5G2
ATP5G3
IB: COX IV
WT KD

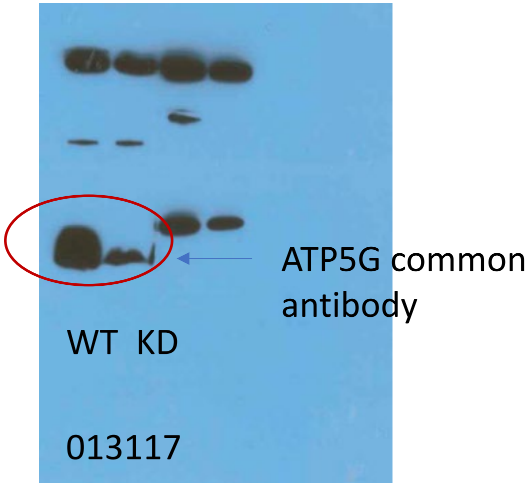
ATP5G common antibody
WT KD
013117

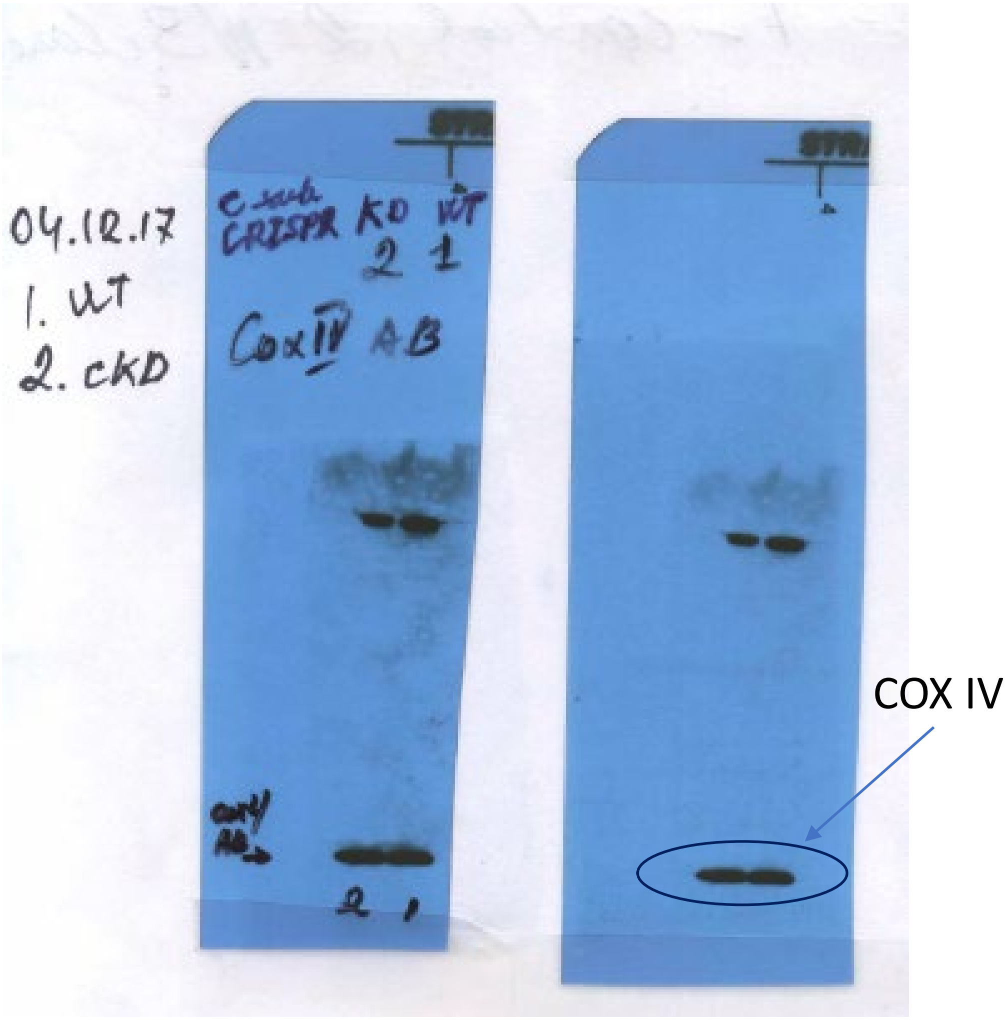
04.12.17
1. WT
2. cKD
KO WT
2 1
CoxIV AB
2 1
COX IV

Figure S1 A.

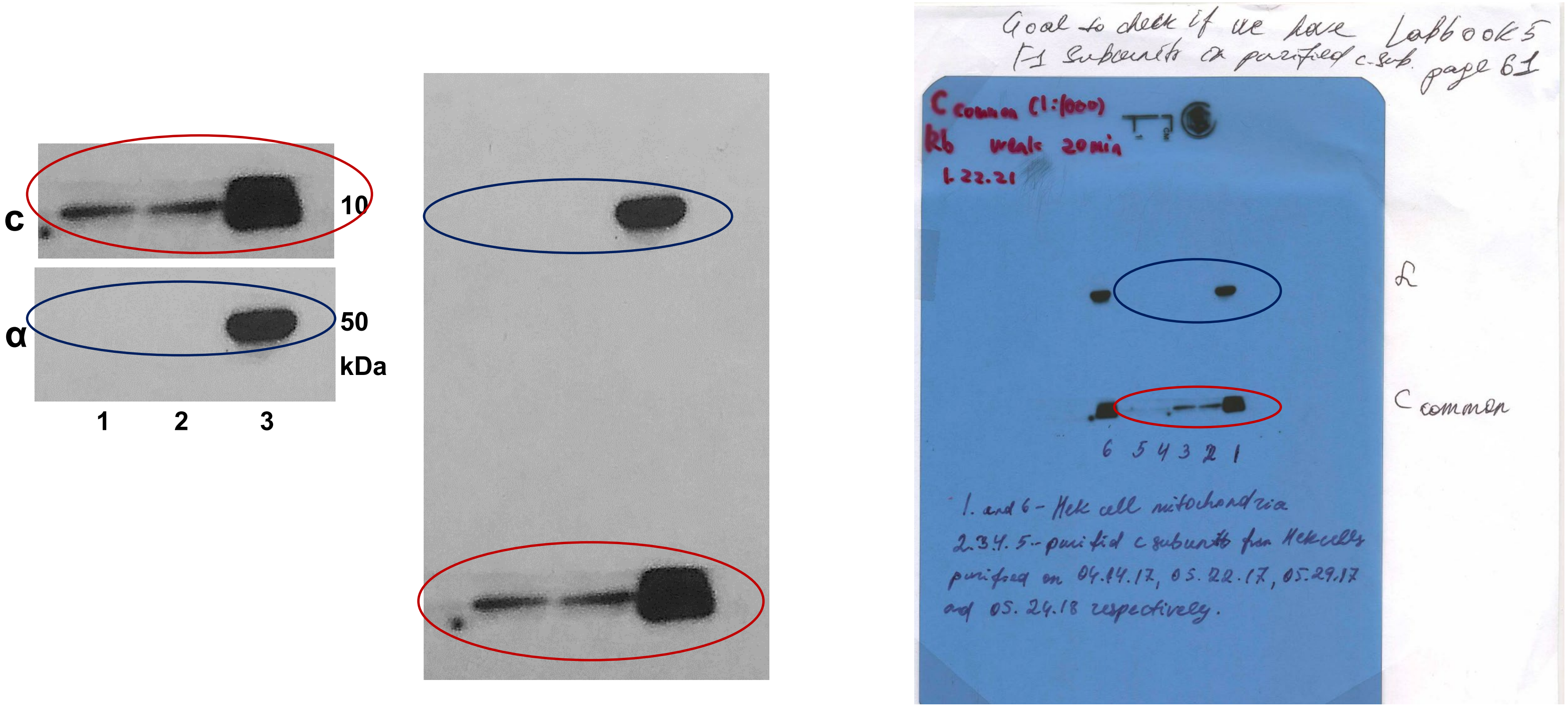
c
10
α
50
kDa
1
2
3
Goal to check if we have F1 subunits or purified c-sub.
Labbook 5 page 61
C common (1:1000)
Rb weak 20 min
1.22.21
6 5 4 3 2 1
C common
1. and 6 - Hek cell mitochondria
2.3.4.5 - purified c subunits from Hek cells
purified on 04.14.17, 05.22.17, 05.29.17
and 05.24.18 respectively.

Fig. S1 D

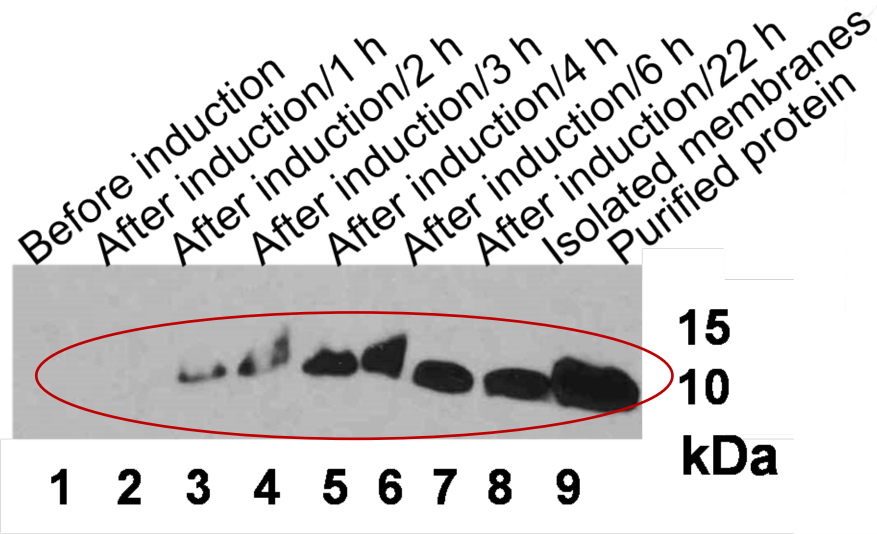
Before induction
After induction/1 h
After induction/2 h
After induction/3 h
After induction/4 h
After induction/6 h
After induction/22 h
Isolated membranes
Purified protein
15
10
kDa
1 2 3 4 5 6 7 8 9

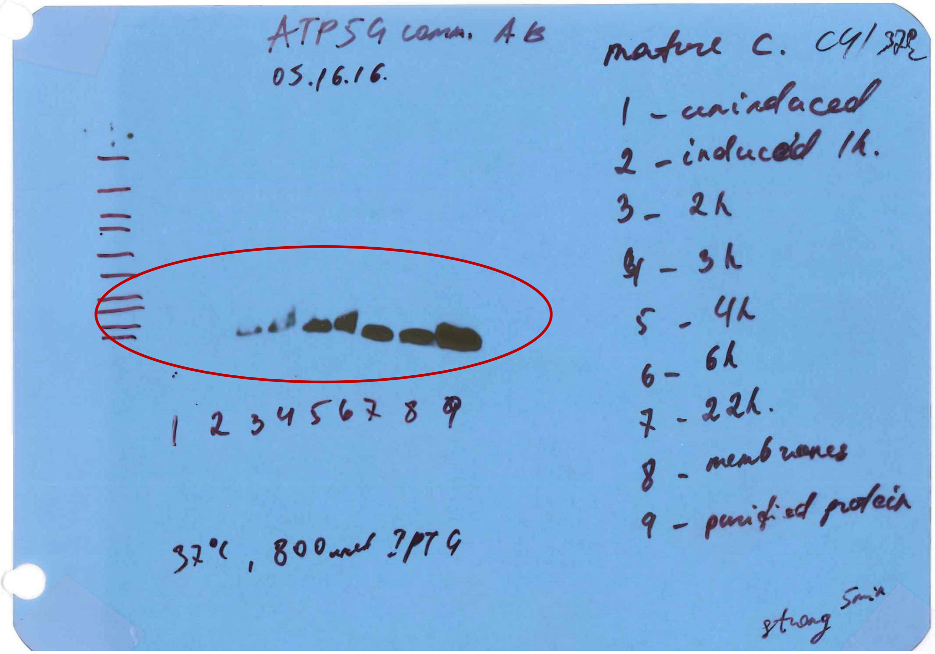
ATP5G comm. AB
05.16.16.
mature c.
1 - uninduced
2 - induced 1h.
3 - 2h
4 - 3h
5 - 4h
6 - 6h
7 - 22h.
8 - membranes
9 - purified protein
1 2 3 4 5 6 7 8 9
37°C, 800 mM IPTG

Fig S1 E

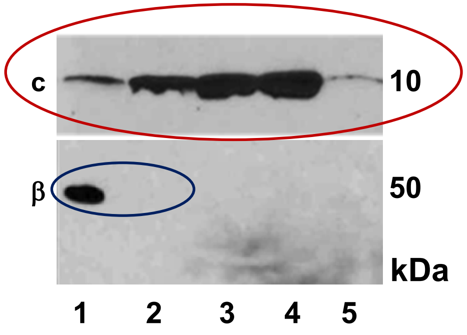

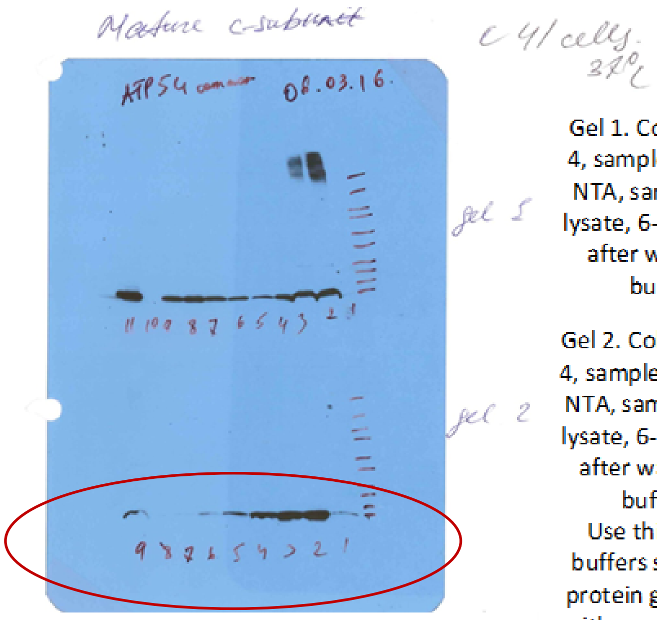

Gel 1. Column 1: 1-4, samples after Ni-NTA, sample 5 cell lysate, 6-11 samples after wash with buffers.

Gel 2. Column 2: 1-4, samples after Ni-NTA, sample 5 cell lysate, 6-9 samples after wash with buffers.
Use this set of buffers since less protein got eluted with wash buffers.

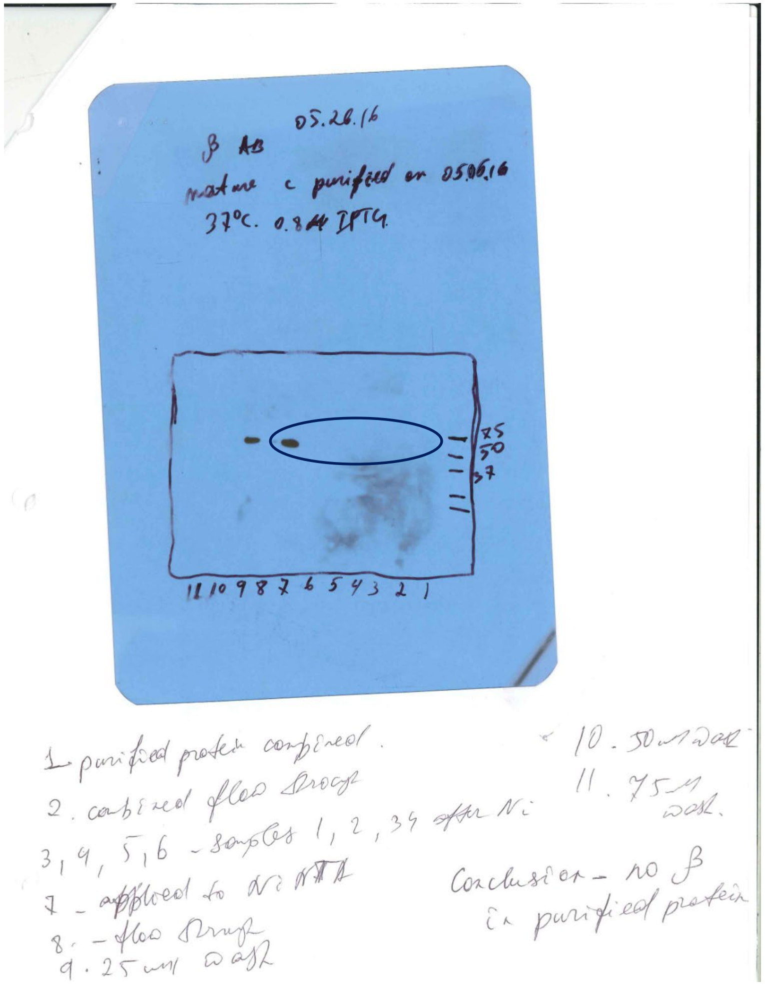

Fig. S3A

A.

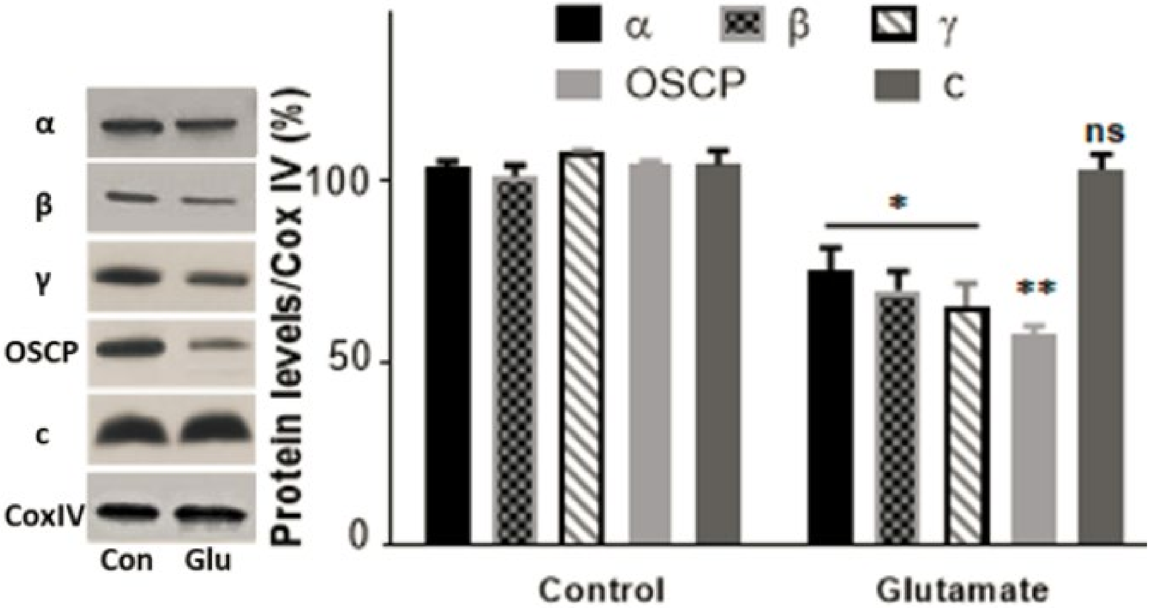
α
β
γ
OSCP
c
CoxIV
Con
Glu
Protein levels/Cox IV (%)
100
50
0
α
β
γ
OSCP
c
ns
*
**
Control
Glutamate

Fig. S3A

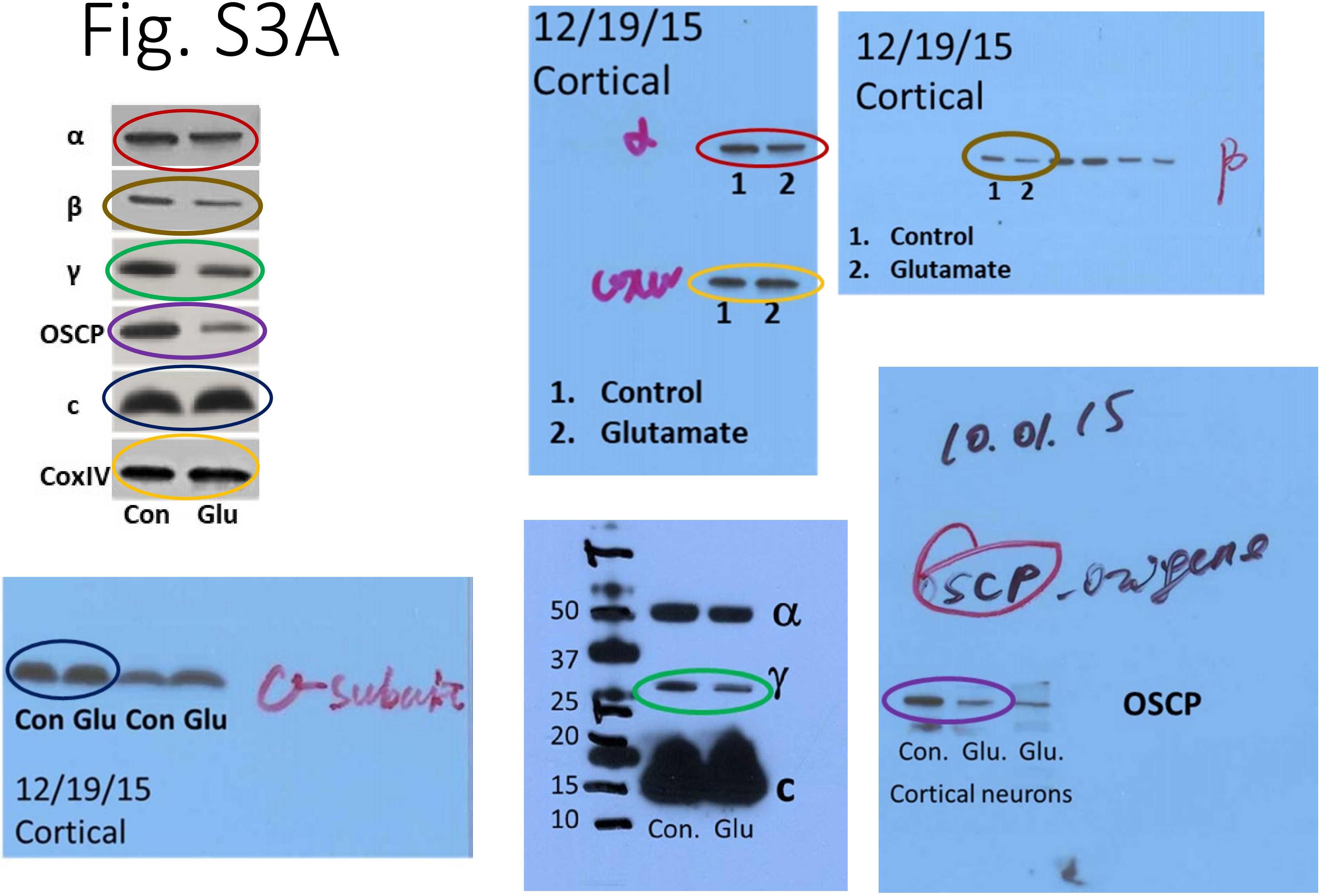

α
β
γ
OSCP
c
CoxIV
Con Glu
12/19/15
Cortical
1 2
1. Control
2. Glutamate
12/19/15
Cortical
1 2
1. Control
2. Glutamate
10. 01. 15
OSCP
Con. Glu. Glu.
Cortical neurons
Con Glu Con Glu
12/19/15
Cortical
50
37
25
20
15
10
α
γ
c
Con. Glu

Figure S2 A

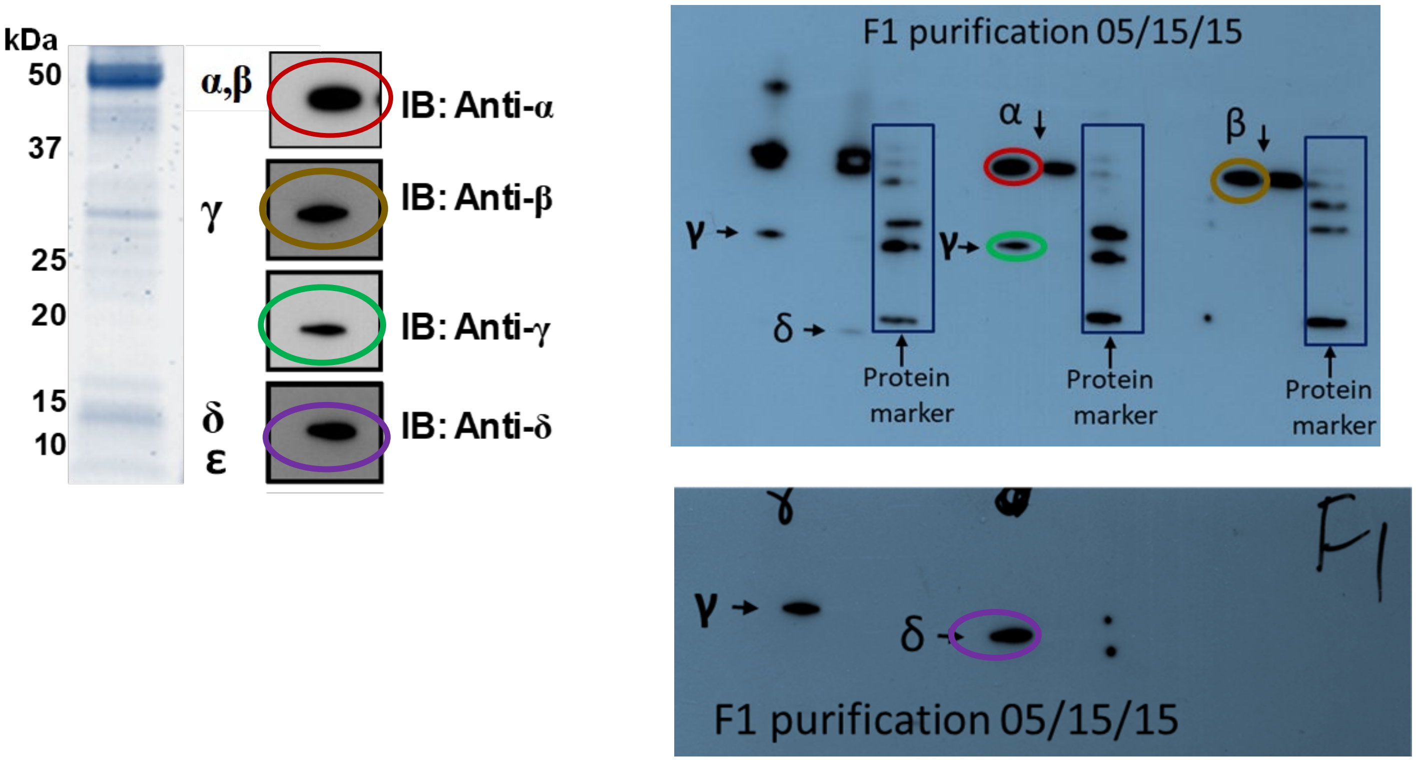
kDa
50
37
25
20
15
10
α,β
γ
δ
ε
IB: Anti-α
IB: Anti-β
IB: Anti-γ
IB: Anti-δ
F1 purification 05/15/15
α
β
γ
δ
Protein marker
Protein marker
Protein marker
γ
δ
F1
F1 purification 05/15/15

Figure S4 C

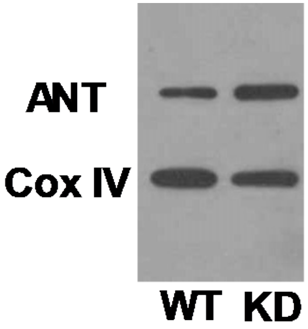

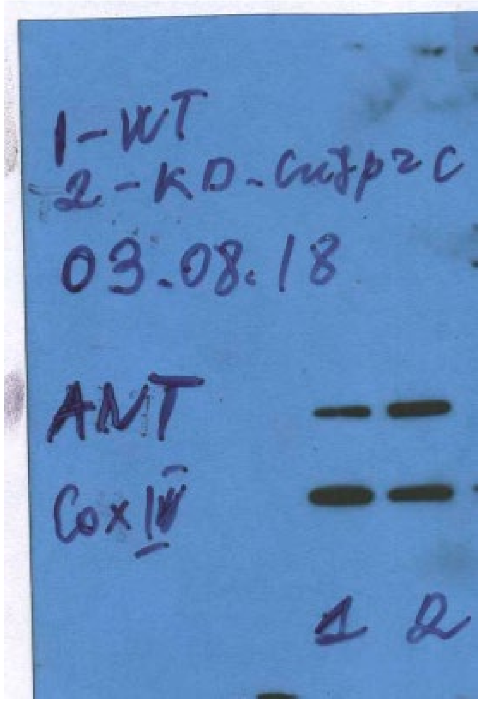

Supplement: Supplementary file 6 — Original data [file 41418_2022_972_MOESM6_ESM.pdf]
